# Genome resequencing clarifies phylogeny and reveals patterns of selection in the toxicogenomics model *Pimephales promelas*

**DOI:** 10.7717/peerj.13954

**Published:** 2022-08-25

**Authors:** Katy E. Klymus, Robert A. Hrabik, Nathan L. Thompson, Robert S. Cornman

**Affiliations:** 1 U.S. Geological Survey, Columbia Ecological Research Center, Columbia, MO, USA; 2 Missouri Department of Conservation, Perryville, MO, USA; 3 U.S. Geological Survey, Fort Collins Science Center, Fort Collins, CO, USA

**Keywords:** Fathead minnow, Pimephales, Genome resequencing, Natural selection, Taxonomy, Titin, MTORC1

## Abstract

**Background:**

The fathead minnow (*Pimephales promelas*) is a model species for toxicological research. A high-quality genome reference sequence is available, and genomic methods are increasingly used in toxicological studies of the species. However, phylogenetic relationships within the genus remain incompletely known and little population-genomic data are available for fathead minnow despite the potential effects of genetic background on toxicological responses. On the other hand, a wealth of extant samples is stored in museum collections that in principle allow fine-scale analysis of contemporary and historical genetic variation.

**Methods:**

Here we use short-read shotgun resequencing to investigate sequence variation among and within *Pimephales* species. At the genus level, our objectives were to resolve phylogenetic relationships and identify genes with signatures of positive diversifying selection. At the species level, our objective was to evaluate the utility of archived-sample resequencing for detecting selective sweeps within fathead minnow, applied to a population introduced to the San Juan River of the southwestern United States sometime prior to 1950.

**Results:**

We recovered well-supported but discordant phylogenetic topologies for nuclear and mitochondrial sequences that we hypothesize arose from mitochondrial transfer among species. The nuclear tree supported bluntnose minnow (*P. notatus*) as sister to fathead minnow, with the slim minnow (*P. tenellus*) and bullhead minnow (*P. vigilax*) more closely related to each other. Using multiple methods, we identified 11 genes that have diversified under positive selection within the genus. Within the San Juan River population, we identified selective-sweep regions overlapping several sets of related genes, including both genes that encode the giant sarcomere protein titin and the two genes encoding the MTORC1 complex, a key metabolic regulator. We also observed elevated polymorphism and reduced differentation among populations (F_ST_) in genomic regions containing certain immune-gene clusters, similar to what has been reported in other taxa. Collectively, our data clarify evolutionary relationships and selective pressures within the genus and establish museum archives as a fruitful resource for characterizing genomic variation. We anticipate that large-scale resequencing will enable the detection of genetic variants associated with environmental toxicants such as heavy metals, high salinity, estrogens, and agrichemicals, which could be exploited as efficient biomarkers of exposure in natural populations.

## Introduction

Fathead minnow (*Pimephales promelas*) is a key model of vertebrate toxicology in aquatic environments, and standardized tests of acute and chronic toxicity in larvae and adults have been developed to support regulatory science (*e.g.*, Benoit, Puglisi & Olson, 1982; Pickering, 1988). The reference genome has recently been updated to further support the integration of transcriptomic, proteomic, and epigenetic methods in fathead minnow toxicology (Martinson et al., 2021). These approaches can help improve the sensitivity and accuracy of toxicological screens, as well as our understanding of molecular modes of action (Ankley & Villeneuve, 2006).

This wealth of toxicology data contrasts with a dearth of population- and evolutionary-genomic study of the fathead minnow and its relatives. Yet natural genetic variation and evolutionary history can inform toxicology in several ways, as highlighted by extensive work in killifish (genus *Fundulus*). For example, because genetic background can be an important modulator of many traits, including toxicity (Whitehead et al., 2010; Whitehead et al., 2017; Crawford et al., 2020), this potential source of variation would be important to consider in toxicological assessments (Baird & Barata, 1998). Genetic-background effects may be particularly strong in inbred research strains (*e.g.*, Brown et al., 2012), which can experience both strong genetic drift and directional selection imposed by culture environment and passaging scheme (Latter & Mulley, 1995; Hoffmann et al., 2001). Although there are critical advantages of inbred stocks for phenotypic analysis (Baird & Barata, 1998) and genetic mapping (Jansen, 2004), and genetic-background effects are expected to be smaller for larval phenotypes than for adult phenotypes (Cheverud, Rutledge & Atchley, 1983), the challenge of identifying late-acting, sublethal effects of chronic exposure and relating them to heterogeneous natural populations remains (Denny, 1987; Horzmann & Freeman, 2018). The broader relevance of candidate genes implicated in toxicological research ultimately depends on the evolutionary stability of those genes within functional networks. The degree to which this holds across taxa is best assessed with comparative genomic approaches such as ortholog clustering, comparative transcriptomics, and synteny mapping (Wei et al., 2002). Comparative approaches may be particularly important for cyprinid fishes because polyploidization events can complicate the inference of orthology and functional equivalence (Catchen, Braasch & Postlethwait, 2011; Chen et al., 2019).

Apart from these considerations of how to extrapolate toxicological findings to natural populations and other species, population-genomic variation provides an alternative framework for assessing toxicological effects, one that is increasingly feasible as sequencing technology continues to improve. This complementary approach seeks to identify spatial and temporal patterns of genetic variation statistically associated with the presence or concentration of toxicants in sampled environments. Such findings provide indirect evidence of toxicant bioavailability and organismal effect that can be further validated in the laboratory. For example, strong environmental clines are often associated with genetic structure at functionally relevant loci (*e.g.*, Reid et al., 2016; Weetman et al., 2018), and allow additional loci or pathways to be discovered (*e.g.*, Ferrero-Serrano & Assmann, 2019). These landscape-level associations between toxicants and genotypes may be particularly useful when captive populations fail to predict short- or long-term impacts in the wild, for example when behavioral mechanisms allow populations to avoid high concentrations of a pollutant (Larrick et al., 1978). Importantly, a high-resolution spatial and temporal record of population-genetic variation in fathead minnow and its congeners can potentially be extracted from the many thousands of specimens currently residing in museum collections (*e.g.*,  http://www.fishnet2.net/) Collections often span decades and document both the native range of the species and its many introductions elsewhere.

Here we report an initial exploration of genetic variation within *P. promelas* and among its congeners. We particularly sought to evaluate low-coverage resequencing of ethanol-preserved specimens as a means of identifying adaptive genetic variation, applied to a single test population introduced to the San Juan River sometime prior to 1950 (Nico, Fuller & Neilson, 2017). We also resequenced two samples of each *Pimephales* congener at higher coverage and performed phylogenetic and evolutionary-rate analyses. These efforts support fathead minnow toxicogenomics by clarifying evolutionary histories and patterns, as well as illustrating the utility of resequencing for the discovery of adaptive alleles in this species.

## Materials & Methods

### Samples

The Museum of Southwestern Biology (Albuquerque, New Mexico) provided fin clips or whole fish from 48 individual specimens for destructive sampling (invoice MSB 20-003), of which 24 were consumed for this pilot study. Sample metadata are provided in File S1 and sample locations are mapped in File S2. Sequenced individuals were originally collected from the San Juan River in the southwestern United States between 2009 and 2014 and had been stored in 95% ethanol. We viewed these samples as favorable for testing DNA extraction and sequencing because of their relatively short time in storage and explicit ethanol concentration. Additionally, the population was established within the past century (Nico, Fuller & Neilson, 2017), which we reasoned was sufficient elapsed time for adaptation to a novel environment to have occurred (fathead minnow has a generation time of 1–2 years; Ross, 2001) yet recent enough for selection signatures to still be evident.

### DNA extraction and sequencing

Genomic DNA was extracted from fin clips with the QuickGene Auto12S Nucleic Acid Extraction System and DNA tissue kit (AutoGen, Inc., Holliston, MA, USA) following the manufacturer’s protocol. Sequencing libraries compatible with the Illumina HiSeq platform were generated with the Lotus DNA Library Prep kit (IDT) following manufacturer’s instructions. The concentration and fragment-size distribution of libraries were measured with an Agilent TapeStation. Pooled libraries were shipped to Quick Biology (Pasadena, CA, USA) for a single lane of HiSeq X sequencing, with a target coverage of approximately 4X per sample. Although samples were individually indexed to allow variation in sequencing output to be assessed, this level of coverage does not allow robust genotyping of individual samples and therefore analyses of genetic variation in this population were based on pooled data or the consensus haploid genome sequence. Demultiplexed reads assigned to individual samples were deposited in NCBI BioProject PRJNA807296, whereas the pooled reads (which include reads not successfully demultiplexed) were deposited in NCBI BioProject PRJNA806159. The latter data are termed the “SJR pool” hereafter.

For congener resequencing, we used samples previously collected and identified or made new collections as needed (see File S1 for sample details). New collections were taken under State of Missouri Wildlife Collector’s Permit (#19224) issued to R. Hrabik, with no additional review required under the relevant animal care policies (see article Declarations for details). Samples were either stored in ethanol or frozen prior to use, and were shipped cold to Admera Health BioPharma Services (South Plainfield, NJ, USA) for DNA extraction and library preparation using the KAPA Hyper Prep kit. Two lanes of 150-bp paired-end sequencing were produced on the HiSeq X platform, multiplexed with three samples per lane, for a target coverage of approximately 20X per sample. Raw sequence data for *Pimephales* congeners has been deposited in NCBI under BioProject PRJNA806850.

For comparison with the SJR pool, we also downloaded short-insert Illumina data (SRR1304883, release date June 10, 2014) upon which the original reference genome was based (FHM 1.0; (Burns et al., 2016). This was one of two technical-replicate accessions available for BioSample SAMN02418978, an inbred pool reared at a DuPont facility. As the FHM 1.0 assembly was generated primarily by the US Environmental Protection Agency (EPA), we refer to these data as the “EPA pool” hereafter.

### Read processing and mapping to reference genome

For the museum specimens, we experienced a relatively high rate of index error in the form of unexpected index combinations as well as index reads that were shifted by one base, limiting the number of demultiplexed reads assigned to samples. For analyses of factors affecting library success, we assigned read pairs to a sample if the forward and reverse index sequences differed by at most one edit distance each from an expected combination. However, for population-genetic analysis, we mapped all reads as a single pool so that reads unattributed to a specific individual could still be included in the population pool.

All read pairs were trimmed with the bbmap package (https://jgi.doe.gov/data-and-tools/bbtools/), specifying a minimum quality of 10 and kmer values for matching exogenous adapters of 15 (internal), 11 (3′ terminal), and 9 (5′ terminal). Reads less than 140 nt after trimming were discarded. FastQC (https://github.com/s-andrews/FastQC) was used to evaluate sequence quality and composition before and after trimming.

The updated and annotated reference genome, EPA_FHM_2.0 (Martinson et al., 2021), was made available by staff of the EPA prior to submission to NCBI. While a parallel gene annotation has since been computed by NCBI using the “GNOMON” pipeline (https://www.ncbi.nlm.nih.gov/genome/annotation_euk/gnomon/) and official scaffold accessions generated (see accession GCF_016745375.1), we continue to use the original EPA scaffold names (which are searchable at NCBI Entrez: https://www.ncbi.nlm.nih.gov) and EPA annotation coordinates for predicted protein sequences.

To analyze factors affecting library quality of museum-archived samples, reads were mapped in pairs with bowtie2 v.2.3.4.2 (Langmead & Salzberg, 2012) using the “end-to-end” and “very-sensitive” parameter switches. Likely PCR duplicates were marked with Picard (https://broadinstitute.github.io/picard/), and read-mapping statistics were computed with the flagstat and stats commands of samtools v. 1.12 (Li et al., 2009).

For population-genetic analysis, all congener samples and the two pools were mapped using bowtie2 in paired mode as already described. A mapping quality filter of 30 was imposed prior to creating multisample “pileup” alignments with the bcftools v. 1.12 (Li et al., 2009) mpileup command, using the recommended map-quality adjust parameter of 50. Biallelic variants were called using bcftools call, and sites with a quality less than the programmatic maximum (*Q* = 999) were excluded. Called indels and single-nucleotide polymorphisms (SNPs) within three positions of a called indel were not used in any analysis. Consensus sequences were generated for each sample with the bcftools consensus command using default parameters, in which called heterozygous sites are resolved in favor of the original reference sequence.

### Mitogenome assembly and phylogeny

To generate a near full-length mitochondrial alignment, we initially mapped reads to the following mitogenome reference accessions: AP012102.1 for *P. vigilax*, AP012101.1 for *P. notatus*, and MG570452.1 (Schroeter et al., 2020) for *P. promelas*. Read mapping was performed with Bowtie2 in paired format with a mapping quality filter of 30, and consensus generation was performed as described above but with a ploidy of one specified. As no reference mitogenome was available for *P. tenellus*, we performed a *de novo* assembly with Spades 3.15.2 (Bankevich et al., 2012), specifying a minimum contig coverage of three and skipping the read-correction step due to the high expected coverage of mitochondrial sequences. Approximately complete mitochondrial genomes were recovered for both *P. tenellus* specimens (accessions OM718773 and OM718774). We elected to repeat this procedure for *P. notatus* as well (accessions OM718772 and OM743017), because the two samples had a mismatch rate of over 3% when mapped to GenBank accession NC_033941. Assembled mitogenomes were annotated with the MitoFish server (Iwasaki et al., 2013).

San Juan River samples were individually confirmed to be *P. promelas* using this consensus-sequence approach, although four samples lacked sufficient coverage for this assessment. Of these 20 consensus sequences, 18 were invariant whereas two had unique and relatively divergent haplotypes indicating multiple introductions. Nonetheless, all 20 consensus sequences had >99% identity to *P. promelas* accessions in GenBank.

Reference mitogenomes for the minnow family Leucisidae were downloaded from NCBI (download date 06/04/21) and appended with the new mitochondrial sequences we had generated. These were then aligned at the nucleotide level with mafft v.7480 (Katoh et al., 2002). We partitioned the alignment five ways: (1) all predicted paired rRNA positions, (2) all predicted unpaired rRNA positions, (3) first codon positions, (4) second codon positions, and (5) third codon positions. All other positions, including tRNAs, noncoding positions and a few dual-frame coding positions were excluded. Coding-sequence coordinates were obtained by submitting accession AP011273.1 to the MITOS annotation server (Bernt et al., 2013), whereas rRNA fold predictions were obtained using the RNAWebSuite server (http://rna.tbi.univie.ac.at/cgi-bin/RNAWebSuite/RNAfold.cgi). A majority-rule consensus phylogeny was produced with MrBayes v.3 (Ronquist & Huelsenbeck, 2003) specifying the general time-reversible substitution model and the “invgamma” model of rate variation. The input and output files of the MrBayes analysis are provided in File S3. To evaluate the consistency of the topology recovered by the Bayesian approach, we also generated maximum-parsimony and maximum-likelihood trees in MegaX v. 10.0.5 (Kumar et al., 2018) using the same alignment. The maximum likelihood analysis also used the general time-reversible model with a gamma + invariant distribution of rate heterogeneity, with five rate classes estimated by the software rather than specified by the user.

### Evolutionary rate analysis

Annotated coding sequence was extracted from each consensus sequence with *gffread* v. 0.12.7 (Pertea & Pertea, 2020) using the longest (or else first-listed) transcript of each gene. Note that codons with unidentified bases (N’s) are ignored in evolutionary rate analysis. We first used the PAML package (v. 4.9j; Yang, 2007) to test for positive selection by fitting alignments to two models with codeml: one that specifies two site-specific evolutionary rates (an estimated *ω*_0_ <1 and a fixed *ω*_1_ = 1) and one that allows a third evolutionary rate *ω*_2_ than can exceed one (positive diversifying selection). The recommended test statistic is twice the difference in log-likelihood of the two models, which is then compared to the *χ*^2^ distribution with two degrees of freedom (Yang, 2007). Because two consensus sequences were available for each species, we randomly grouped these into two four-species alignments and ran codeml on both, using an unrooted tree topology consistent with our nuclear species tree (see Results). We did not include all eight sequences together in the PAML analyses because initial analyses with intraspecific sequences included yielded implausibly high test statistics, indicating that gene trees with very short branches gave biased parameter and likelihood estimations. We therefore considered genes to have significant excess nonsynonymous substitution only if both four-species alignments had a false discovery rate (FDR)-corrected *P*-value ≤ 0.05. We excluded short alignments (less than 100 codons) and also those that had elevated transition ratios (parameter *κ* ≥ 5), because strong transition bias complicates rate estimation (Yang & Nielsen, 2000). A single alignment with a very long tree length was excluded as well, as this implies excessive substitutions or a read-mapping artifact. Genes identified as undergoing positive selection by PAML were then further tested using the related BUSTED method (Murrell et al., 2015) of the HyPhy package v. 2.5.32 (Kosakovsky-Pond & Muse, 2005). We used all eight sequences in BUSTED analyses because the method does not impose branch and site-specific rates but instead uses a random-effects model to determine the overall likelihood of an unlocalized episode of positive selection. That likelihood statistic is also compared to a *χ*^2^ distribution with two degrees of freedom (Murrell et al., 2015).

To further validate genes identified by these two methods to be under positive selection in *Pimephales*, we assessed whether positive selection was still supported with the inclusion of additional related cyprinids in each alignment. These expanded alignments included inferred orthologs from zebrafish (*Danio rerio,* Howe et al., 2013), *Cyprinus carpio* (Xu et al., 2014), *Carassius auratus* (Chen et al., 2019), *Sinocyclocheilus rhinocerous* (Yang et al., 2016), and *Puntigrus tetrazona* (synonymous with *Puntius tetrazona*; assembly accession GCF_018831695). These species were chosen based on their high ranking in BLASTP (Camacho et al., 2009) search results of *P. promelas* predicted proteins as well as the availability of RefSeq models predicted computationally from relatively complete reference genomes. The top protein RefSeq match for each of these species was assumed to be orthologous and the corresponding RefSeq mRNA extracted and aligned, deleting poorly aligned regions if necessary. If the expanded BUSTED test was not significant, we also ran the branch-specific method aBSREL (Smith et al., 2015) to confirm that at least one *Pimephales* branch gave evidence of positive selection.

Enrichment of gene ontology (GO) annotations in gene sets was tested with goSeq v. 4.1.1 (Young et al., 2010), which is designed to incorporate length bias in gene sets. Length bias was a potential concern because the power to estimate evolutionary rate classes should generally increase with coding sequence length, which could necessitate a weighting function; however, no relationship was evident. We therefore performed the enrichment test using the standard hypergeometric method that assumes no length bias. To avoid repeated testing of very similar annotation terms, only “cellular compartment” GO categories were used because this category had the lowest *P*-value for an individual term. *P*-values were adjusted using the Benjamini–Hochberg method with the R function *p.adjust* (R Core Team, 2018). The mapping of genes to ontologies was taken from the EPA-submitted annotations (Martinson et al., 2021) by parsing the gff-formatted feature file.

### Variant calling, genetic diversity statistics, and outlier detection

To measure variation within the SJR pool, the average per-site minor allele frequency (MAF) was computed directly from the allele counts reported in the mpileup file, *i.e.*, without regard to whether a site was called a variant or not in the multispecies comparison. For these estimates, we ignored sites with fewer than 15 aligned reads or with only a single variant read. MAF was summed across all nonmajor alleles if more than two alleles were reported at a site. This approach was preferred for this SJR-specific statistic so as not to be limited to sites called from a heterogeneous combination of intraspecific population pools and individual congener samples. However, the called and filtered multisample SNPs were necessarily used to calculate Weir’s *F*_ST_ with VCFtools (Danecek et al., 2011), with each species grouped as a population. The EPA pool was excluded from the *F*_ST_ calculation because it was strongly inbred and thus not indicative of natural variation. Alignment depth was calculated in sliding windows with VCFtools and for bed-formatted exons with samtools.

Statistical packages are available for identifying sliding-window variation in allele frequencies and *F*_ST_ between two population pools (*e.g.*, Kofler et al., 2011; Kofler, Pandey & Schlötterer, 2011; Boitard et al., 2013). However such differences would not be meaningful for understanding adaptation in the SJR pool given the strong but mosaic inbreeding in the EPA pool (see Results). We therefore performed empirical assessments of outlier polymorphism patterns within the SJR pool only, which for individually-genotyped samples is often implemented by defining outlier quantiles of Tajima’s D statistic and nucleotide diversity (*π*) (Feulner et al., 2013; Reid et al., 2017). However, estimation of these statistics from high-throughput sequencing can be fraught, particularly for pooled data with unequal representation of relatively few chromosomes (Tajima, 1983; Futschik & Schlötterer, 2010; Ferretti, Ramos-Onsins & Pérez-Enciso, 2013; Boitard et al., 2013). Interpretation of Tajima’s D is further complicated by demographic factors (Ramírez-Soriano et al., 2008) such as the likely bottlenecks and admixture of the SJR population during colonization (see Results). We therefore followed an empirical approach similar to that of Reid et al. (2016), but rather than using separate outlier cutoffs for MAF and *F*_ST_, we sought to identify consecutive genomic windows that were visual outliers in a two-dimensional plot of MAF and *F*_ST_. We adopted this two-variable approach because allele frequencies and *F*_ST_ at a locus are generally not independent yet modeling their dependence is challenging (Flanagan & Jones, 2017). To minimize stochastic effects on allele-frequency estimation, we averaged MAF over large genomic windows (50 kb with a 25-kb step), required a minimum of 100 sites per window for *F*_ST_, and required consecutive windows to be outliers. Our specific procedure was to first identify candidate scaffolds with multiple windows at the margins of the plotted two-variable distribution, and then to plot those variables linearly along those candidate scaffolds for inspection. Contiguous regions with conspicuous outlier windows were then re-plotted relative to the total distribution of all windows to confirm that they were collectively near the margins of the distribution. Although we considered the two-variable distribution to be more useful for identifying outliers, we nonetheless confirmed that all selective-sweep regions identified by that approach included at least one window in the 1% quantile tails for both MAF and *F*_ST_ (File S4). MAF and *F*_ST_ estimates for all genomic windows are provided in File S5.

In accordance with US Geological Survey policy for data access, primary data associated with this study have also been released in parallel through an approved repository (Cornman et al., 2022).

## Results and Discussion

### Genome coverage from resequencing

Fin clips from ethanol-stored fish yielded variable but generally adequate DNA quality and quantity (File S6). Because we do not expect archived samples to yield sequencing libraries of the same quality as fresh samples, we sought to identify library benchmarks that predicted read-pair mapping and PCR duplication rates. We did not identify any appreciable variation among libraries in rates of PCR duplication, which were modest and averaged 3.9% (File S7). However, we did observe a tendency for lower rates of paired-read mapping to the genome reference when the library DNA concentration was below approximately 3 ng/µl or the peak DNA fragment size was below approximately 450 bp, indicating that these values may be benchmarks for high yield. Peak fragment size was significantly rank-correlated with mapping rate (Spearman’s r of 0.453, *P* = 0.027). We do not suggest that samples yielding libraries below these benchmarks should be avoided, only that such benchmarks can guide the prioritization and relative weighting of samples in resequencing runs. Indeed, we expect that very old or poorly preserved samples will yield relatively fragmented DNA, and also that the relationship between these metrics and sequencing yield will vary among library-preparation protocols. Note that the genome mapping rate is expected to be much less than 100% because the reference sequence is extensively masked, largely due to the high repeat content of the genome (Martinson et al., 2021).

Exon coverage per unit sequencing effort was similar among all congener samples as well as the SJR pool (File S8), averaging 13.8–14.2 read pairs per kilobase per million mapped reads (RPKM) at a mapping quality threshold of 30. Exon coverage in these ranges is expected to be effective for identifying fixed differences among congeners as well as the majority of heterozygous genotypes. Samples from other *Pimephales* species had flatter distributions of coverage and were very similar in shape for the *P. tenellus* pair and the *P. vigilax* pair, whereas the *P. notatus* samples differed somewhat. We attribute the higher frequency of low-coverage exons in congeners to either sequence divergence or paralogy, as both factors reduce mapping quality scores.

### Nuclear and mitochondrial differentiation within Pimephales

**Figure 1:**
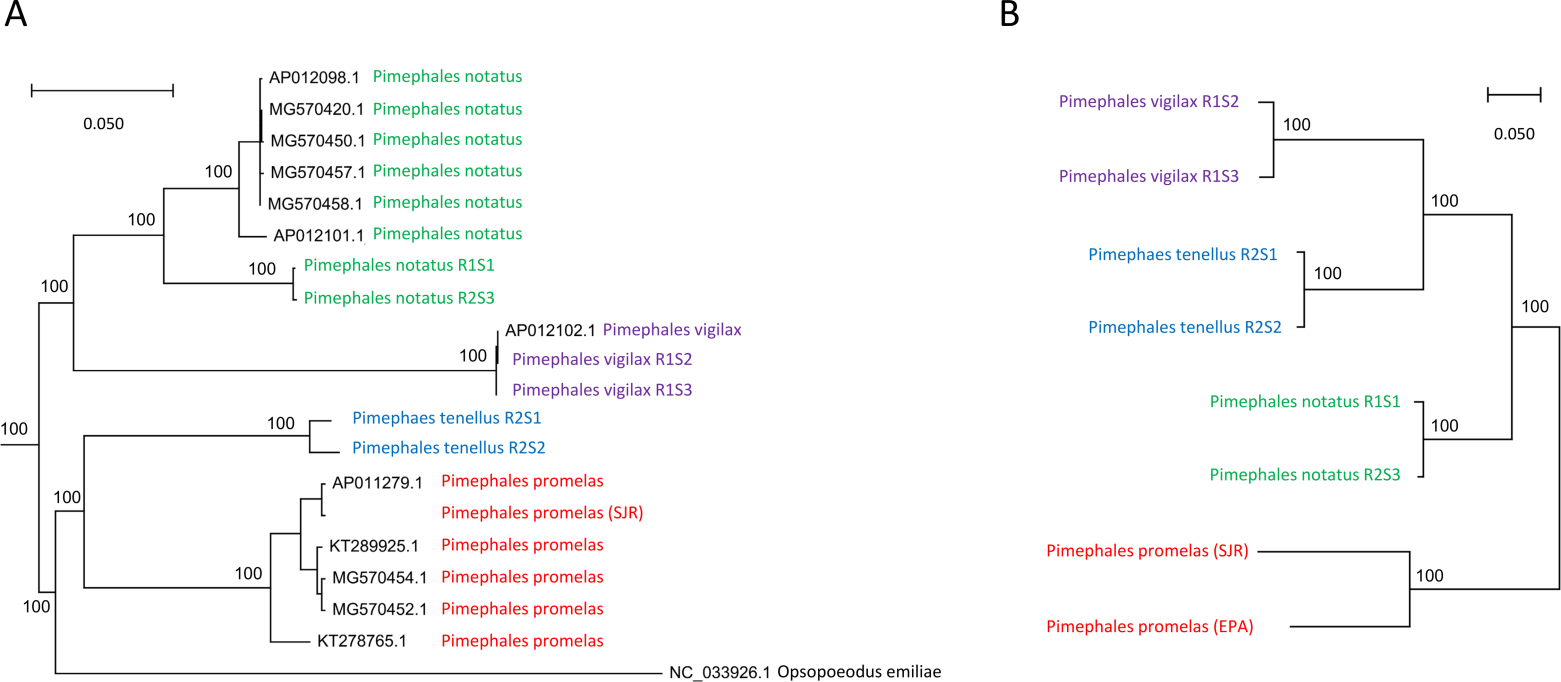
Phylogenetic analysis of *Pimephales* mitochondrial and nuclear variation. (A) Bayesian phylogeny of full mitochondrial ribosomal RNA (rRNA) and protein-coding sequence. For model estimation, rRNA sites were partitioned into paired and unpaired sites, whereas coding sequence was partitioned by codon position. (B) Neighbor-joining phylogeny of nuclear single-nucleotide polymorphisms (SNPs) using the identity-by-state (IBS) distance measure. The *P. promelas* samples are population pools represented as pseudodiploid samples, *i.e.*, variant sites in those pools are generally represented as heterozygotes resulting in exaggerated intraspecific branch lengths.

Eight alignments (six resequenced individuals, the SJR pool, and the EPA pool) were used to ascertain biallelic SNPs. Because the SJR and EPA pools have much higher total coverage and represent a population of chromosomes, SNP ascertainment favors detection of variable sites within those pools. A total of 5,509,510 SNPs were called among the eight samples, of which 4,712,129 were genotyped in at least one congener. The latter SNP set was used to calculate a neighbor-joining phylogeny (Fig. 1) based on average pairwise identity by state (IBS). The SJR and EPA pools were treated as pseudodiploid samples for this analysis so that a pairwise IBS could be calculated (biallelic sites in these pools would typically be represented as heterozygotes). This approach better accommodates intraspecific variation within congener samples, at a cost of distorting the divergence between the two fathead minnow pools. All nodes of the resulting tree had 100% bootstrap support and the topology is consistent with hypothesized relationships based on morphology (reviewed by Schmidt, Dowling & Gold, 1994), specifically that *P. tenellus* and *P. vigilax* are more closely related whereas *P. notatus* is sister to *P. promelas*. Inspection of individual gene trees used in the evolutionary rate analysis (described below), which were based on codon-aware nucleotide substitution models rather than simple differences in state, showed them to be entirely consistent with the IBS tree.

A mitochondrial alignment of over 15,000 positions across five partitions and including numerous outgroups produced a *Pimephales* topology inconsistent with the nuclear tree (Fig. 1) but consistent with the mitochondrial topology of Bielawski, Brault & Gold (2002). Those authors noted the conflict between this mitochondrial topology and several morphological characters, and suggested that those traits may have evolved independently in parallel. Given the amount of data supporting our nuclear phylogeny, we consider interspecific mitochondrial capture to be a more likely explanation of discordance, possibly from *P. promelas* to *P. tenellus*. Hybridization among cyprinids is common, and mitochondrial capture without apparent impact on nuclear genetic relationships has been reported even between relatively distant species pairs (Freyhof et al., 2005). Figure 1 also shows a relatively deep phylogeographic division within *P. notatus* (*P. notatus* accessions in Fig. 1 originated from Atlantic watersheds, if reported, whereas our *P. notatus* samples were midwestern in origin). Overall, these results are in line with other instances of phylogeographic structure and nuclear-mitochondrial discordance reported for *Pimephales* populations (Ballesteros-Nova et al., 2019; Schönhuth et al., 2016).

The full mitochondrial tree showing the inferred relationship of *Pimephales* to other Leucisidae is shown in File S9 . The Bayesian tree placed pugnose minnow (*Opsopoeodus emiliae*) within *Pimephales* with high support, a topology also supported by likelihood and parsimony methods (File S10). Nonetheless, *O. emiliae* represents a long, monotypic evolutionary branch with a novel karyotype (Campos & Hubbs, 1973); its placement could potentially be influenced by long-branch attraction within the larger cyprinid phylogeny or distorted by reticulate evolution, as we hypothesized above for *Pimephales*. Importantly, Blanton, Page & Ennis (2011) recovered a polytomy for *Pimephales* and *Opsopoeodus* with mitochondrial data but their single-locus nuclear data supported a more distant relationship between the two taxa. Additional nuclear data will likely be needed to place *O. emiliae* with confidence.

The two *P. tenellus* samples derive from different subspecific groups, *P. tenellus tenellus* and *P. tenellus parviceps*, that are morphologically and spatially distinct although intergrades also occur (Hubbs & Black, 1947). In both the nuclear and mitochondrial trees, the two *P. tenellus* specimens are not more divergent from each other than are any other *Pimephales* conspecific pair. To investigate whether particular genomic windows exist that are highly diverged between the two subspecies, we re-called intraspecific variants for each conspecific pair separately to minimize ascertainment bias with respect to this question. A histogram of the number of fixed differences between sample pairs in 50-kb genomic windows does not indicate any high-divergence windows between *P. tenellus* subspecies (File S11). We conclude that the two subspecies are not strongly diverged genetically relative to the variation found in comparable sampling of other *Pimephales* species. This result is not inconsistent with strong morphological or ecotypic differentiation, which can accrue from modest allele-frequency shifts across multiple loci, particularly when those loci are in linkage disequilibrium (Latta, 2004). Although we are not able to evaluate this possibility with our current data, resequencing of *P. tenellus* populations may identify patterns of allele-frequency divergence and linkage disequilibrium that differentiate morphological types.

### Evolutionary rate analysis

We identified 11 genes with evidence of positive selection on coding sequence under both the PAML and BUSTED models (FDR-corrected *P* < 0.05; Table 1). Most significant alignments were more than 400 codons long and had comparable levels of nucleotide substitution across the species tree (Table 1). We repeated nine of the eleven tests with expanded alignments that included other cyprinids (see Materials and Methods) to test the consistency of the finding while allowing that positive selection may have also occurred among related taxa (two genes were too divergent in other cyprinids to test). BUSTED found significant evidence of positive selection in eight of the nine expanded alignments, with the remaining gene *nebulin* significant only under the branch-specific method implemented in aBSREL. Increasing the number of taxa, and thus the data available for rate estimation, strengthens the conclusion that these genes have been targets of positive selection. Although the multiple methods used here increase our confidence in these results, important caveats should be noted. First, because genes are not independently annotated in the fathead minnow congeners, we cannot confirm that the true ortholog is actually present in all species or that reads deriving from close paralogs are not also mapping. Second, substitutions are presumed to have fixed along the phylogeny post-divergence, yet retained ancestral polymorphisms and lineage-sorting events may be relatively common among recently diverged species (*e.g.*, Pollard et al., 2006).

**Table 1:** Genes with significant evidence of positive selection within *Pimephales*. *P*-values for *Pimephales*-only alignments are Benjamini-Hochberg corrected for false discovery rate (FDR). A second FDR correction was not imposed on the expanded alignments. *P*-values for *Pimephales*-only alignments are Benjamini-Hochberg corrected for false discovery rate (FDR). A second FDR correction was not imposed on the expanded alignments.

Gene (transcript)	Annotated name	Description	PAML Tree1 *P*-value	PAML Tree2 *P*-value	BUSTED *P*-value	Minimum alignment length	Minimum tree length (substitutions per codon)	Expanded alignment test and *P*-value^**^
FMg011803 (FMt022778)	tmc8^*^ (tmc7)	Transmembrane channel-like protein 7	0.022	0.046	0.001	675	0.087	Not tested
FMg009899 (FMt019254)	*plxnc1*	Plexin-C1 (semaphorin protein receptor)	0.010	0.001	0.010	1404	0.080	B: 3.675E−7
FMg025023 (FMt045894)	*nlrc6*	NLR family CARD domain-containing 6	0.008	0.015	0.010	933	0.008	B: 1.21E−3
FMg011037 (FMt021169)	*tnfaip2b*	Tumor necrosis factor, alpha-induced protein 2b	0.011	0.003	0.025	667	0.102	B: 1.29E−2
FMg006019 (FMt011957)	(unnamed)	Nebulin	0.028	0.011	0.025	6055	0.058	a: 3.22E−2
FMg014331 (FMt027393)	*adgre3*	Adhesion G protein-coupled receptor E3	0.001	0.000	0.027	612	0.090	B: 2.45E−5
FMg002767 (FMt005485)	*alcamb*	Activated leukocyte cell adhesion molecule b	0.005	0.030	0.032	558	0.115	B: 0
FMg016591 (FMt031555)	*cfbl*	Zgc:158446 protein (complement factor b-like)	0.001	0.023	0.032	1200	0.101	B: 1.75E−4
FMg010614 (FMt020395)	*scn3b*	Sodium channel, voltage-gated, type III, beta	0.040	0.017	0.039	218	0.086	B: 1.24E−3
FMg007724 (FMt015056)	*wasf3b*	Wiskott-Aldrich syndrome protein family member 3b (WAVE3 homolog)	0.018	0.023	0.039	464	0.058	B: 4.90E−2^***^
FMg008000 (FMt015520)	uncharacterized	SEA-domain containing	0.012	0.026	0.039	427	0.136	Not tested

**Notes.**

* Annotated gene name is *tmc8* but is putatively orthologous to *tmc7* of *Danio rerio* based on homology search.

** *P*-values computed with BUSTED are marked with “B,” whereas *P*-values computed with aBSREL are marked with “a”.

*** *Sinocyclocheilus rhinocerous* removed from expanded alignment due to higher sequence divergence.

At least eight of the eleven positively selected genes encode membrane-associated proteins (Table 1), a high ratio given that their overall proportion in most proteomes is ≤ 30% (Krogh et al., 2001). Nonetheless, no cellular-component ontology terms were significantly enriched after FDR correction. Among these membrane-associated genes was the sodium transporter *scn3b*, which was also identified as a target of positive selection in the cyprinid *Gymnocypris przewalskii* (Tong & Li, 2020). *G. przewalskii* inhabits high-salinity environments in central Asia, and *scn3b* was one of several solute transporters implicated in salt tolerance in that species. In *G. przewalskii*, *scn3b* is not gill-associated but rather expressed in a variety of tissues at modest levels (Tong & Li, 2020). A second membrane transporter gene, annotated as *tmc8* but most similar by protein sequence to *tmc7* in other vertebrates, is one of a family of well-conserved transmembrane-channel proteins of unknown function. The *tmc7* gene was one of many associated with growth-rate variation in common carp (Su et al., 2018).

Two of the eleven positively selected genes have known immune functions: *cfbl* encodes a complement system component (Zhang & Cui, 2014) and *adgre3* encodes a surface receptor expressed by various myeloid immune cells (Hamann et al., 2016). The *nlrc6* gene is a member of the diverse NLR gene family that contributes to pathogen recognition in innate immunity (Hu et al., 2014; Sahoo, 2020). However, the NLR-C subfamily is highly expanded and variable in copy number in fish (Laing et al., 2008), such that it is difficult to ascribe functions to individual gene-family members. Two selected genes have been implicated in axon guidance during development: *alcamb* is important in guiding retinal ganglion cells in *D. rerio* (Diekmann & Stuermer, 2009) whereas the semaphorin-receptor *plxnc1* can function in axon guidance but may also participate in other developmental processes (Jongbloets & Pasterkamp, 2014). The w*asf3b* gene is a paralog of mammalian *wasf3*/*wave3*, which regulates the cytoskeletal mechanics of cell migration (Kansakar et al., 2020), notably during the development of retinal photoreceptor cells (Corral-Serrano et al., 2020). We speculate that positive selection on both *alcamb* and *wasf3b* reflects adaptation in retinal development, as fish are known to display extreme variation in retinal morphology and spectral tuning (Kusmic & Gualtieri, 2000). The gene *tnfaip2b* is believed to have roles in exocytosis and membrane conformation (Kimura, 2018). The gene *nebulin* encodes a muscle microfiber protein important in muscle structure, contraction, and force generation (Yuen & Ottenheijm, 2020). The final selected gene encodes a predicted protein with multiple domains that are associated with extracellular-matrix glycosylation and cleavage (“SEA” domains, Palmai-Pallag et al., 2005), but is otherwise uncharacterized.

### Selective-sweep candidate regions

We compared polymorphism patterns within the EPA and SJR pools to determine if sliding-window tests of differentiation between the two would be appropriate, knowing that the EPA pool had been inbred prior to sequencing (Burns et al., 2016). This comparison confirmed that the EPA pool was highly inbred overall but also highly mosaic in terms of average MAF within sliding windows, with regions of very low polymorphism intermixed with regions of near normal polymorphism (File S12). Although we cannot determine from a single inbred pool whether this mosaicism is entirely due to chance or also reflects instances of heterosis (higher fitness associated with heterozygosity), resequencing independent inbred lines should distinguish those possibilities, which could be important for understanding sources of genetic variance in toxicogenomic assays.

**Figure 2:**
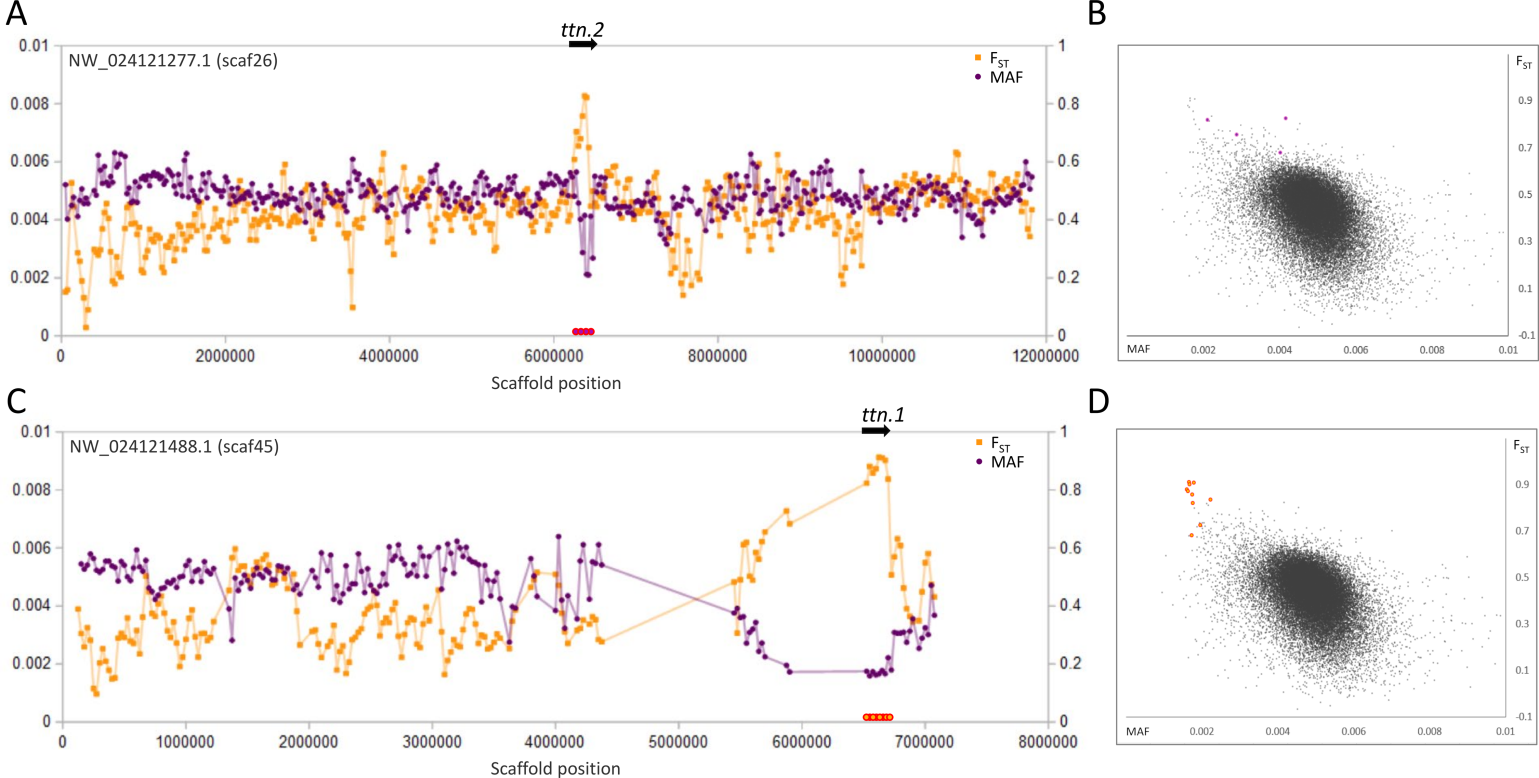
Genetic variation consistent with selective sweeps in the vicinity of fathead minnow (*Pimephales promelas*) genes *ttn.1* and *ttn.2*. (A) Variation in minor allele frequency (MAF) of the San Juan River pool and among-species F_ST_ for scaf26, with the location of *ttn.2* marked. Each point represents values within a 50-kb genomic window (sliding, with a step of 25 kb) and is placed at the first coordinate of the window. Absent points indicate too few variants were detected for estimation (see text) and usually reflect repeat-masked regions. Colored circles on the horizontal axis denote windows that appear as outliers in (B). (B) Distribution of MAF and F_ST_ for all genomic windows analyzed, with the points marked in (A) correspondingly colored. (C) Variation in MAF and F_ST_ for scaf45, with the location of *ttn.1* marked. (D) Values of genomic windows marked in (C) relative to the distribution of values for all genomic windows.

**Figure 3:**
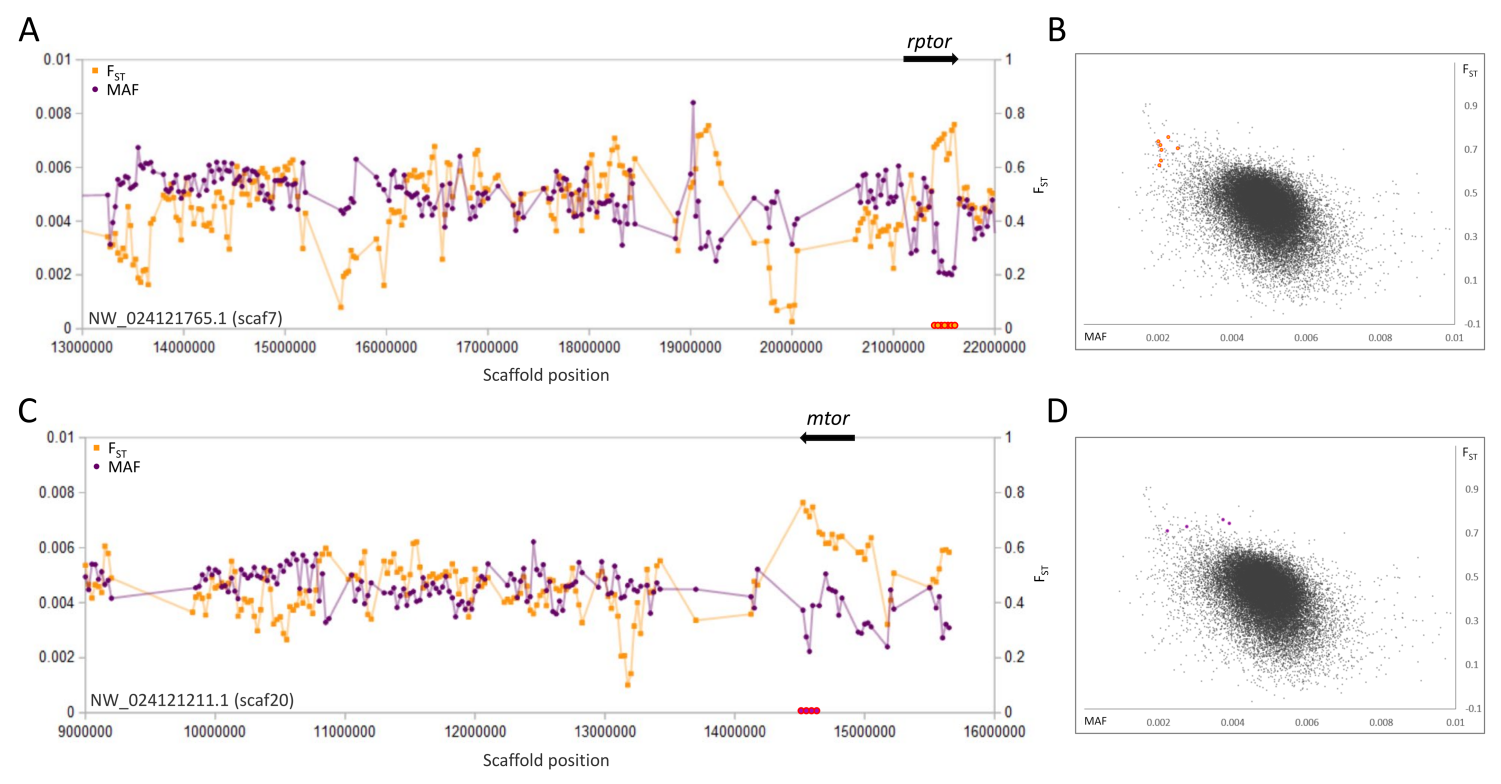
Genetic variation consistent with selective sweeps in the vicinity of fathead minnow (*Pimephales promelas*) genes *mtor* and *rptor*. (A) Variation in minor allele frequency (MAF) of the San Juan River pool and among-species F_ST_ for scaf7, with the location of *rptor* marked. Each point represents values within a 50-kb genomic window (sliding, with a step of 25 kb) and is placed at the first coordinate of the window. Absent points indicate that too few variants were detected for estimation (see text) and usually reflect repeat-masked regions. Colored circles on the horizontal axis denote windows that appear as outliers in (B). (B) Distribution of MAF and F_ST_ for all genomic windows analyzed, with the points marked in (A) correspondingly colored. (C) Variation in MAF and F_ST_ for scaf20, with the location of *mtor* marked. (D) Values of genomic windows marked in (C) relative to the distribution of values for all genomic windows.

**Figure 4:**
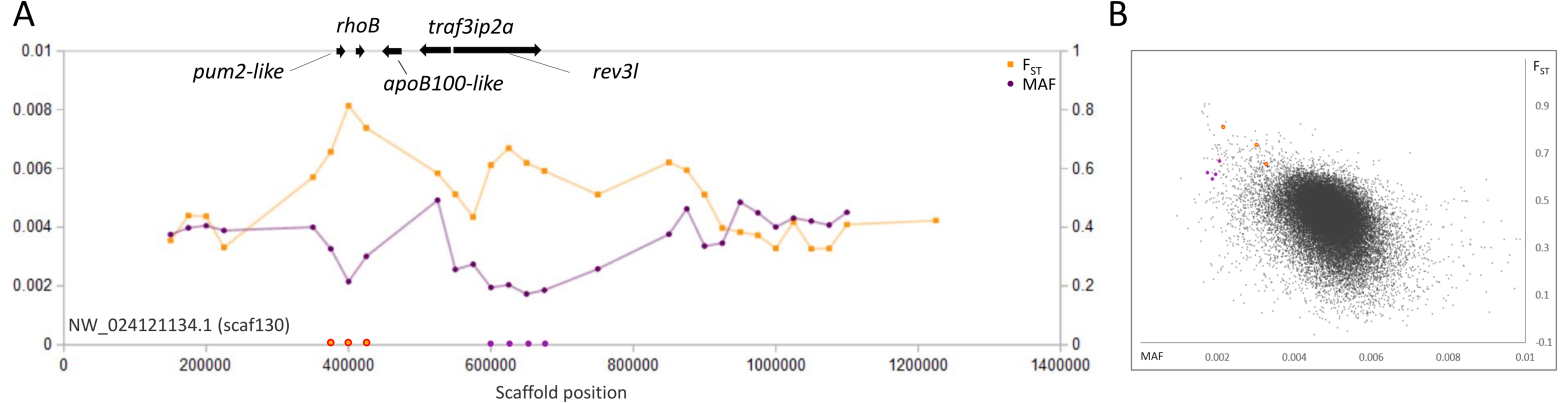
Genetic variation consistent with selective sweeps in the vicinity of fathead minnow (*Pimephales promelas*) genes *rhoB* and *rev3l*. (A) Variation in minor allele frequency (MAF) of the San Juan River pool and among-species F_ST_ for scaf130, with the location of genes near outlier windows marked. Each point represents values within a 50-kb genomic window (sliding, with a step of 25 kb) and is placed at the first coordinate of the window. Absent points indicate that too few variants were detected for estimation (see text) and usually reflect repeat-masked regions. Colored circles on the horizontal axis denote windows that appear as outliers in (B). *rhoB* and *rev3l* are the only annotated genes present within the strongest outlier of each set of consecutive windows. (B) Distribution of MAF and F_ST_ for all genomic windows analyzed, with the points marked in (A) correspondingly colored.

**Figure 5:**
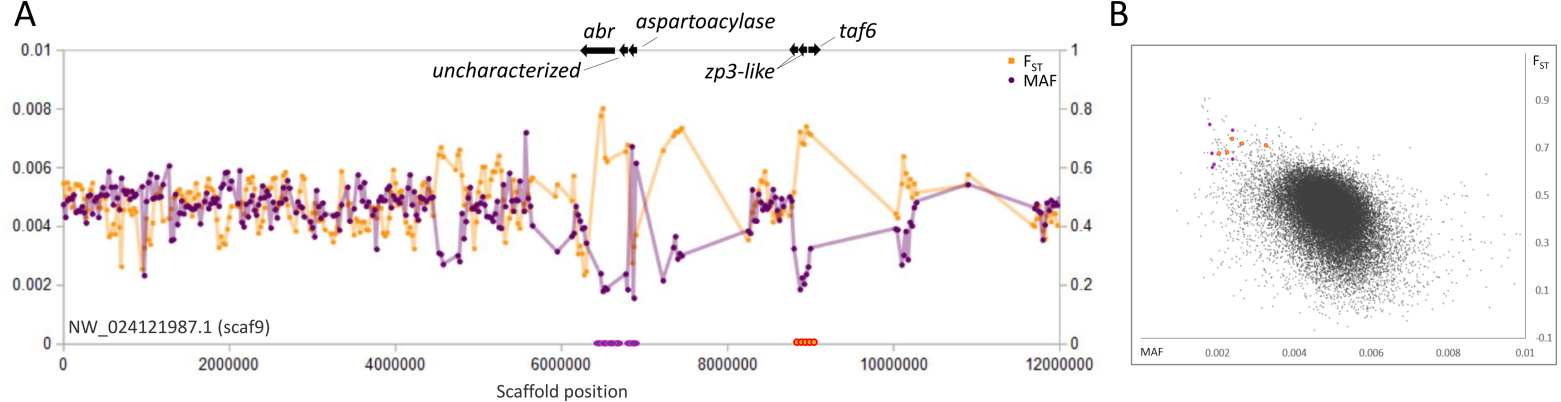
Genetic variation consistent with selective sweeps on scaf9 of the fathead minnow (*Pimephales promelas*) genome assembly. (A) Variation in minor allele frequency (MAF) of the San Juan River pool and among-species F_ST_, with the location of genes near outlier windows marked. Each point represents values within a 50-kb genomic window (sliding, with a step of 25 kb) and is placed at the first coordinate of the window. Absent points indicate that too few variants were detected for estimation (see text) and usually reflect repeat-masked regions. Colored circles on the horizontal axis denote windows that appear as outliers in (B). (B) Distribution of MAF and F_ST_ for all genomic windows analyzed, with the points marked in (A) correspondingly colored.

As the skewed polymorphism in the EPA pool renders it unsuitable as a reference for expected background variation in the SJR pool, we used an empirical approach to identify candidate regions under selection in the latter. We argue that consecutive genomic windows that have high *F*_ST_ among congeners and low polymorphism within the SJR pool are consistent with a selective sweep, provided they are sufficiently strong outliers relative to the overall distribution of these values across all genomic windows. Several strong examples of this pattern were found that are both physically clustered and strongly coincident with gene boundaries (Figs. 2–5). Moreover, three cases involved pairs of functionally related genes, providing independent evidence of their biological relevance.

The most pronounced outliers were coincident with the two titin-encoding genes, *ttn.1* and *ttn.2* (Fig. 2), which occur on separate scaffolds in the fathead minnow assembly but are tandemly arrayed in *D. rerio*. Titin is a large, modular protein that helps structure the muscle sarcomere and has spring-like properties, influencing the elasticity of muscle fibers. Many different isoforms may be expressed among muscle types and developmental stages (Wang et al., 1991; Steffen et al., 2007).

A second pair of selective sweeps coincided with the genes *rptor* and *mtor* (Fig. 3), the products of which form the key regulatory heteromer MTORC1 (Liu & Sabatini, 2020). These genes lie on different scaffolds and in both cases *F*_ST_ is highest and polymorphism lowest at their 3′ends (both genes exceed 100 kb in length and have a concentration of 3′exons). Although other nearby genes cannot be excluded as potential targets of selection, the strong overlap of sweep windows with *rptor* and *mtor*, coupled with their well-documented physical and functional interaction, strongly indicates that they are driving the sweep pattern.

A third example is a pair of outlier regions on scaffold 130, in which the sweep pattern peaks in two 50-kb windows containing the single genes *rhoB* and *rev3l*, respectively (Fig. 4). Although we again cannot rule out the possibility of selection on other nearby genes, functional similarities between *rhoB* and *rev3l* further supports their relevance because both genes function in the repair of genomic DNA lesions. Gene *rev3l* encodes the catalytic subunit of DNA polymerase zeta, which supports translesion replication (Jansen et al., 2009), whereas *rhoB* functions in double-strand-break repair (Mamouni et al., 2014). Both genes can be induced by various types of environmental DNA damage (*e.g.*, Fritz & Kaina, 1997; Lin et al., 2016) and promote the repair of UV-induced lesions (Fritz, Kaina & Aktories, 1995; Jansen et al., 2009). However, they also have regulated expression in normal development (Lange et al., 2016; Vega & Ridley, 2018) and *rev3l* is essential for embryonic viability in mice (Lange et al., 2016). UV exposure in the San Juan River Basin is in fact higher than in the native range of fathead minnow (Fioletov et al., 2004), but whether increased exposure is related to the signatures we identified remains to be tested.

A fourth scaffold, scaf9, contains two distinct sweep regions that do not appear functionally related (Fig. 5). The first region contains the 5′ end of the *abr* gene, a general regulator of rho GTPases (Chuang et al., 1995), the uncharacterized gene LOC120472185, and an aspartoacylase-encoding gene (LOC120472146). Aspartoacylase expression is a known transcriptomic biomarker of insecticide exposure in fish (Beggel et al., 2012; Stinson et al., 2022). The second region includes two homologs of zona-pellucida sperm-binding protein 3 (*zp3*) as well as the basal transcription factor *taf6*. ZP3 proteins are documented to evolve rapidly and have been targets of positive selection in diverse taxa including fish (Turner & Hoekstra, 2008; Calkins, El-Hinn & Swanson, 2007; Ugwu et al., 2018).

At present, we are not able to test what environmental factors may be associated with the selective sweeps we observed, as we have not sampled across environmental clines and do not know the progenitor populations. However, titin loci have been implicated in adaption to environmental variables in other fish species. Whitehead et al. (2010) identified distinct titin expression patterns in polychlorinated biphenyl (PCB)-tolerant killifish that may have a cardioprotective role. Milano et al. (2014) identified a titin locus as one of several genes containing outlier SNPs consistent with adaptive divergence in European hake, and polymorphism frequencies were correlated with temperature and salinity in some populations. It is also possible that the selective sweeps on titin genes and MTORC1 genes reflect selection on body morphology in novel flow conditions. For example, Cureton & Broughton (2014) found repeated evolution of body shape in *P. vigilax* after changes in streamflow, and similar intraspecific associations between body shape and flow regime have been reported in other cyprinids (Haas, Heins & Blum, 2015; Jacquemin & Pyron, 2016; Kern & Langerhans, 2018). Adaptive genetic variation associated with the sarcomere proteins titin and nebulin could potentially contribute to such rapid morphological adaptation by altering muscle elasticity. Furthermore, MTORC1 has been shown to be essential for translating experienced mechanical stress into muscle growth (Goodman, 2013; You et al., 2019). Alternatively, *mtor* has been hypothesized to act as an evolutionarily labile modulator of how nutrition interacts with growth, development, and body shape (Valente et al., 2013), thereby contributing to rapid morphological evolution. This hypothesis has been suggested by several studies implicating *mtor* in ecotype differentiation (Macqueen et al., 2011), sexual differentiation (Manor et al., 2015), and domestication (Seiliez et al., 2008; Skiba-Cassy et al., 2009) in fish.

### Genomic regions of high polymorphism and low F_ST_

**Figure 6:**
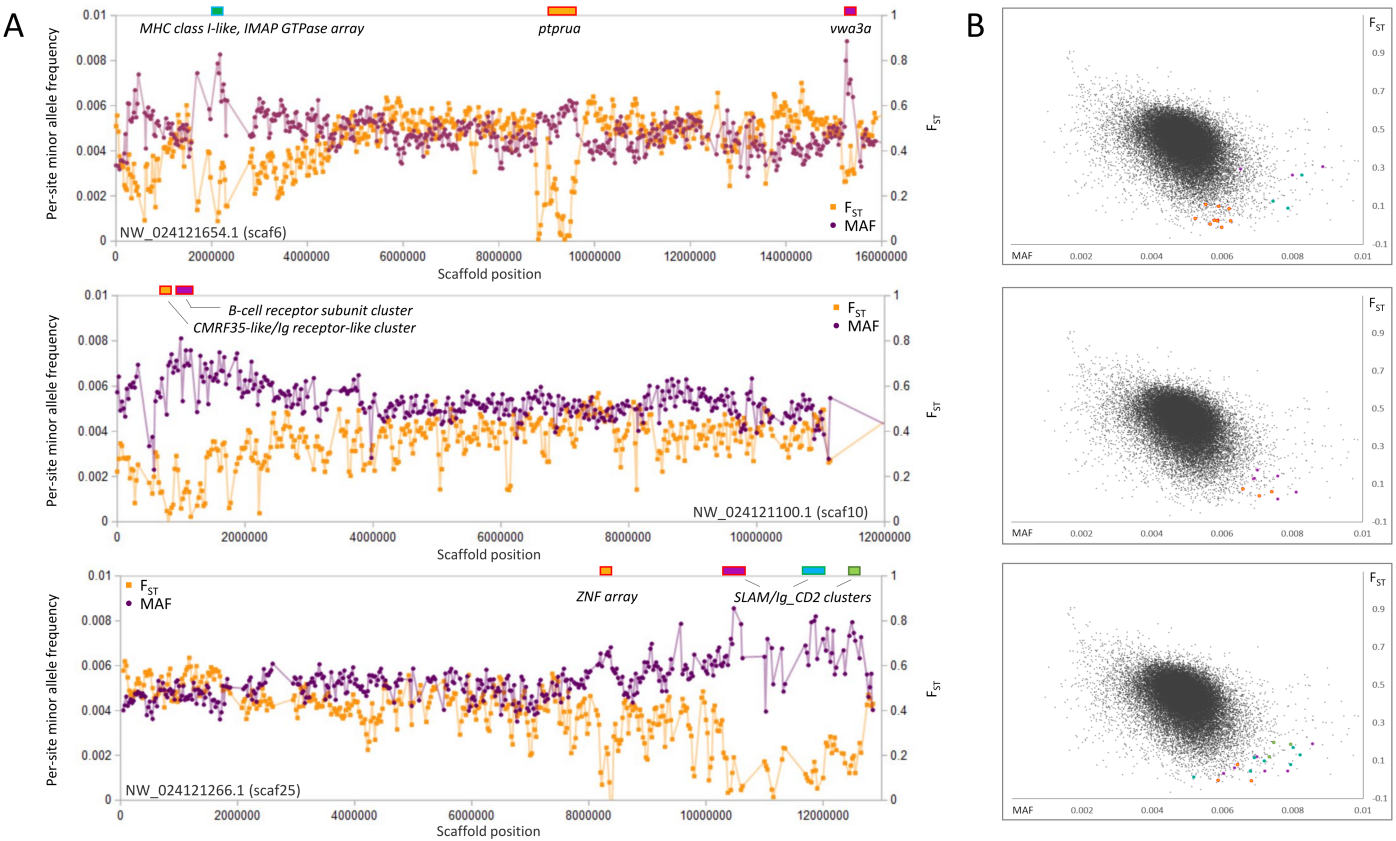
Genes and gene clusters associated with windows of high intraspecific variation and low among-species F_ST_. (A) Variation in minor allele frequency (MAF) of the San Juan River pool and among-species F_ST_ on three scaffolds, with selected genes or clusters of genes marked. Each point represents values within a 50-kb genomic window (sliding, with a step of 25 kb) and is placed at the first coordinate of the window. Points are absent if fewer than 100 variants were called in the window. Colored boxes on the horizontal axis denote locations of windows that appear as outliers in (B). (B) Distribution of MAF and F_ST_ for all genomic windows analyzed, with the points marked in (A) correspondingly colored.

In contrast to selective sweeps, consecutive genomic windows with unusually low *F*_ST_ among species and high polymorphism within the SJR pool are candidate regions for balancing or frequency-dependent selection (Fijarczyk & Babik, 2015). By inspection (Fig. 6), clustered outlier genomic windows exhibiting this pattern are often not associated with specific genes but rather arrays of homologous immune genes known to evolve rapidly by both nonsynonymous substitution and copy number. Examples include a cluster of multifamily B-cell receptor subunits (Clark & Giltiay, 2018) on scaf10 and a cluster of MHC class I (Shum et al., 2001) and IMAP GTPase genes (Huang et al., 2019) on scaf6. Additional immune-gene clusters with similar outlier polymorphism patterns include distinct clusters of SLAM5-like homologs (Cannons, Tangye & Schwartzberg, 2011) on scaf25 and a number of CMRF35-like homologs near the B-cell receptor array on scaf10. CMRF35-like genes regulate aspects of inflammation, including eosinophil accumulation in mucosa (Moshkovits et al., 2014) and activation of microglial (Ejarque-Ortiz et al., 2015) and myeloid cells (Shik et al., 2014). All three multigene immune clusters marked in Fig. 6 are associated with a breakdown in synteny relative to zebrafish (File S14), further demonstrating the high rate of structural evolution associated with these regions. Coordinates and descriptions of genes and gene clusters identified in Figs. 2–6 are detailed in File S15.

The overall association that regions of high polymorphism in the SJR pool and low *F*_ST_ among species have with immune gene clusters is striking, yet some exceptions are evident in Fig. 6. An array of ten zinc-finger (ZNF) proteins on scaf25 lacks annotated immune functions but does show strong evidence of recent paralogy: six of the ten proteins cluster together phylogenetically rather than with homologous ZNF proteins identified by BLASTP in the genomes of other cyprinids (File S16). The genes have dissimilar exon structures and several are physically overlapped, indicating that they are not retrogenes or tandem duplicates. A second outlier region on scaf6 coincides with the gene *ptprua*, a conserved single-copy ortholog based on annotation provided on the NCBI gene page (https://www.ncbi.nlm.nih.gov/gene/563584) and conserved synteny with the corresponding zebrafish region. The *ptprua* gene encodes a receptor-linked protein tyrosine phosphatase, which as a family have diverse roles in signal transduction, although the PTPRU protein uniquely lacks tyrosine phosphatase activity and instead inhibits the activity of other family members via substrate competition (Hay et al., 2020). A third outlier region on scaf6 is coincident with the *vwa3a* gene, which encodes a cell–surface glycoprotein of unknown function, although the family-defining von Willebrand factor domain is generally found in extracellular matrix proteins and often those involved in cell adhesion (Whittaker & Hynes, 2002). We found MAF to be elevated in two of the three scaf6 regions in the EPA pool as well (File S17), with a strong, narrow spike associated with *vwa3a* but no elevated polymorphism in the vicinity of *ptprua*. This pattern supports the hypothesis of overdominance or frequency-dependent selection at *vwa3a*, manifested even in a captive inbred population.

Although the low *F*_ST_ and high diversity in these regions could reflect the retention of relatively ancient haplotypes by overdominance or frequency-dependent selection, as has been shown for immune genes in human (Hughes & Nei, 1989; Slade & McCallum, 1992) and fish (Criscitiello et al., 2004), the pattern could also be an artifact of paralogy *per se*. That is, the incorrect mapping of reads from recently derived paralogs unrepresented in the reference genome should elevate perceived polymorphism and, if those paralogs are shared among multiple species, reduce perceived *F*_ST_. Haplotype phasing of individuals with long-read sequencing (*e.g.*, Kuleshov et al., 2014) could help disentangle these possibilities. Given the prominence of copy-number variable gene families coincident with these regions, we hypothesize that copy-number variation is the stronger driver of these polymorphism patterns overall, whereas overdominance or frequency-dependent selection more likely explains patterns around the conserved single-copy genes *ptprua* and *vwa3a*. However, a third possible contributor to this polymorphism pattern is sex-associated polymorphism. Sex-associated loci are known to be dispersed in the zebrafish genome, and polymorphisms closely linked to loci involved in sexual differentiation can be maintained at intermediate allele frequencies (Anderson et al., 2012; Wilson et al., 2014). This phenomenon may account for the outlier polymorphism pattern associated with the ZNF array on scaf25, as a cluster of similar ZNF genes are annotated on zebrafish chromosome 4 between 60 and 61 Mb (*Ensembl* names *Znf1109*, *Znf1033*, *Znf1080*, *Znf1121*, *Znf1130*, and *Znf1044*) and this genomic window has been found by two separate studies to contain SNPs strongly associated with sex in that species (Anderson et al., 2012; Wilson et al., 2014).

### Limitations and caveats

The biological relevance of the selective sweep regions we identified is supported by functional overlap between physically unlinked sweep regions, and our approach further validated by the detection of patterns in the vicinity of immune gene clusters similar to what has been reported in killifish (Reid et al., 2017) and other species (Hughes & Nei, 1988; Hughes & Nei, 1989; Slade & McCallum, 1992; Criscitiello et al., 2004; Angata, 2006). Nonetheless, we acknowledge that our current data are not suitable for explicit statistical tests of allele-frequency divergence or linkage disequilibrium relative to a reference population. The detection of selective sweeps can also be complicated by demographic history, including bottlenecks and introgression, as well as the genetic architecture of selected traits (Storz, 2005). Although our detection of divergent mitochondrial haplotypes indicates multiple introductions of fathead minnow to the San Juan River, the population is relatively isolated, no major dispersal barriers are apparent within the sampled watershed, and the species has a high growth rate and short generation time (Ross, 2001). We therefore do not expect strong genetic structure at neutral nuclear loci in this basin, but this could be confirmed with diploid genotyping.

Although we imposed rigorous thresholds for per-site coverage and total SNPs per window (see Materials and Methods), we nonetheless confirmed that these candidate sweep regions were not associated with atypical levels of sequence coverage that might indicate a mapping artifact (File S13). Although the two titin genes appear to have somewhat elevated coverage relative to the mean, this likely reflects the much lower level of gapped or masked sequences in those windows due to their very long coding sequences (masked sequence is typically intergenic or intronic). Inspection of coverage patterns for titin exons relative to intron or flanking sequences did not indicate any duplication of annotated exons, and the predicted gene architectures are similar to their orthologs in zebrafish.

## Conclusions

In this study, we used shotgun resequencing to assess genetic differentiation across the genome for phylogenetic analysis and to detect signatures of natural selection. Our results clarified some evolutionary relationships within *Pimephales*: we found little nuclear or mitochondrial differentiation between morphologically distinct subspecies of *P. tenellus*, whereas mitochondrial data indicated a deep division within *P. notatus*. However, the overall mitochondrial topology of *Pimephales* conflicted with that of nuclear SNPs, implying past mitochondrial capture and undermining the phylogenetic utility of mitochondrial sequences in the genus. An additional result of our genus-level analysis is the identification of eleven genes with excess nonsynonymous substitution (positive selection). While there is no expectation that these genes should overlap with selective sweep regions in the SJR population, it is noteworthy that they included *nebulin*, the product of which interacts directly with titin in structuring sarcomeres. Sequencing other close relatives would be expected to improve evolutionary rate estimates for these genes and allow the timing of selection to be better localized. Including *O. emiliae* in such an effort would be expected to simultaneously clarify the phylogenetic placement of that species.

SNP variation within and among *Pimephales* species revealed several probable selective sweeps in the San Juan River population. We speculate that the prominence of titin genes and MTORC1 subunits in unlinked sweep regions reflects mechanisms of morphological and metabolic adaptation to new environments, consistent with a body of other work. We anticipate that an analysis of independent invasions of similar watersheds by fathead minnow would illuminate any parallelisms, as has been done in other species (Fang et al., 2020; Whitehead et al., 2017), which could inform this hypothesis. Identifying commonalities of and constraints on local adaptation during the invasion of novel habitats may help inform the management of longer-lived and more recently bottlenecked invaders such as carp species in North America.

Detection of probable selective sweeps in the SJR population is an important proof-of-principle for using archived samples to detect adaptive alleles associated with toxicants of concern, as many potential toxicological stressors of natural *Pimephales* populations will likely have arisen over comparable time scales. Future work could seek to leverage museum archives associated with documented spatial and temporal clines in pollutants such as industrial hydrocarbons, heavy metals, environmental estrogens, agrichemicals, and salinity. This landscape approach could be extended to model potential responses of cyprinids to climate change, by examining standing variation associated with environmental variables most responsive to climate (Gautier, 2015). Once candidate haplotypes are identified that are strongly associated with an environmental variable of interest, quantitative PCR assays can be designed to estimate their frequency relative to control markers. Although frequency estimates can be derived for DNA pooled from archived samples or new collections, we ultimately expect the most efficient approach to quantifying contemporary allele-frequencies will be from environmental DNA samples. Conceptually similar eDNA genotyping assays have already been applied to diverse management needs, such as determining the proportion of conspecific carp derived from a non-native source (Uchii, Doi & Minamoto, 2016), noninvasive genotyping of cultured oysters (Holman et al., 2019), and noninvasive haplotyping of whale sharks (Dugal et al., 2022).

##  Supplemental Information

10.7717/peerj.13954/supp-1 Supplemental Information 1 Sample metadata Sample metadata and identifiers assigned by the National Center for Biotechnology Information (NCBI) that link to deposited data.

10.7717/peerj.13954/supp-2 Supplemental Information 2 Map of original sample collections within the San Juan River, United States Map generated with the National Map Viewer tool (https://apps.nationalmap.gov/viewer/) using coordinates provided by the Museum of Southwestern Biology, Albuquerque, New Mexico.

10.7717/peerj.13954/supp-3 Supplemental Information 3 Data for mitochondrial phylogeny Input and output files in NEXUS format for the mitochondrial genome phylogeny.

10.7717/peerj.13954/supp-4 Supplemental Information 4 Scatterplot of minor allele frequency (MAF) and F_ST_ for all 50-kb genomic windows passing coverage thresholds Windows in the 1% or 99% quantiles of both distributions are marked red.

10.7717/peerj.13954/supp-5 Supplemental Information 5 Minor allele frequency (MAF) and FST in consecutive 50-kb genomic windows with 25-kb step For the San Juan River pool of fathead minnow samples, MAF was calculated for all sites with a minimum depth of 15 and at least two reads supporting a variant. FST was calculated for windows with at least 100 called variants, with samples of each Pimephales species considered a separate population. Only windows with estimates for both parameters are shown.

10.7717/peerj.13954/supp-6 Supplemental Information 6 Associations between characteristics of individual San Juan River samples of *Pimephales promelas* and sequencing mapping rate to the reference genome (A) Year of sample collection. (B) Library peak fragment size. (C) Extracted DNA concentration.

10.7717/peerj.13954/supp-7 Supplemental Information 7 Sequencing library characteristics, paired-end mapping rates and inferred PCR duplication rates for shotgun resequencing of museum specimens

10.7717/peerj.13954/supp-8 Supplemental Information 8 Exon coverage per unit sequencing effort for samples used in evolutionary rate analysis Horizontal axis represents length-normalized coverage in reads per kilobase per million mapped reads (RPKM). Vertical axis represents the number of exons in each bin of RPKM.

10.7717/peerj.13954/supp-9 Supplemental Information 9 Full Bayesian phylogeny of 92 Leucisidae mitochondrial genomes ¡!–[if !supportAnnotations]–¿¡!–[endif]–¿The basal node of *Pimephales* plus *Opsoepodu* s is circled in orange. Branches for each Pimephales species are colored as shown in the legend. ¡!–[if !supportAnnotations]–¿¡!–[endif]–¿

10.7717/peerj.13954/supp-10 Supplemental Information 10 Mitochondrial topologies recovered by other methods Phylogenetic topologies recovered for the 92-genome alignment with non-Bayesian methods are identical to the Bayesian tree with respect to the placement of *Pimephales* and *Opsopoeodus*. (A) Maximum parsimony phylogeny, and (B) Maximum likelihood phylogeny. See Methods for analysis parameters.

10.7717/peerj.13954/supp-11 Supplemental Information 11 Histogram of fixed differences between conspecific pairs of *Pimephales* in 50-kb genomic windows

10.7717/peerj.13954/supp-12 Supplemental Information 12 ¡!–[if !supportAnnotations]–¿¡!–[endif]–¿Comparison of average minor allele frequencies (MAF) within the San Juan River (SJR) and US Environmental Protection Agency (EPA) population pools for 50-kb genomic windows (see Methods for pool descript (A) Histogram of average MAF for SJR genomic windows with the mean value of all windows marked by the red line. (B) Histogram of average MAF for EPA genomic windows with the mean value of all windows marked by the red line. (C) Population MAF in individual genomic windows ordered by position on scaf1, the longest scaffold in the *Pimephales promelas* assembly, revealing a dichotomous pattern of relatively high or very low MAF in the EPA pool in contiguous genomic regions.

10.7717/peerj.13954/supp-13 Supplemental Information 13 Sequencing coverage is not atypical in selective sweep regions Depth of mapped reads in non-overlapping 50-kb windows is shown for both *Pimephales promelas* pools for the regions analyzed in Figs. 2–5. Coverage is scaled relative to the median value of all windows, and is expected to vary due to stochasticity, biased capture of extreme nucleotide compositions, and the proportion of masked or ambiguous sequence in a window (which prevents reads from mapping). Gene-rich regions with few gaps should therefore have somewhat higher than median coverage. Note that dots are positioned according to the first coordinate in each window.

10.7717/peerj.13954/supp-14 Supplemental Information 14 Synteny comparisons between the genome assemblies of *Danio rerio* and *Pimephales promelas* in the vicinity of immune gene clusters associated with high polymorphism and low F_ST_ Each axis represents the genomic sequence of the indicated accession and coordinates. Each dot represents protein homology between the two genome regions that exceeds the scoring threshold. Blue dots indicate homology in the same chromosomal orientation and red dots indicate opposite orientations. Ancestral synteny is indicated by contiguous points forming an approximately diagonal line, inversions as reversals in the orientation and color of the line, and loss of synteny as unordered pattens of pairwise homology or absence of homology. (A–C) show the *P. promelas* regions containing clusters of immune genes identified in Fig. 6 and their best corresponding match in *D. rerio.* Equivalent gene clusters in the two species are approximated by black circles. The green circle in (C) highlights a deletion in the *P. promelas* assembly of a cluster of zinc-finger (ZNF) transcription factors present in *D. rerio*.

10.7717/peerj.13954/supp-15 Supplemental Information 15 Identifiers, descriptions, and coordinates of genes identified in selective-sweep regions and high-polymorphism regions

10.7717/peerj.13954/supp-16 Supplemental Information 16 Gene tree of zinc finger protein transcription factors Gene tree of ten zinc-finger protein (ZNF) transcription factors within the genomic windows marked on scaf25, together with homologs identified in three other high-quality *de novo* genome assemblies within Cyprinidae: *Danio rerio*, *Carassius auratus*, and *Cyprinus carpio*. (An eleventh *Pimephales promelas* ZNF gene is adjacent but not within the marked genomic windows). Six of the ten *P. promelas* genes cluster together with relatively short branches (marked by bracket), indicating gene amplification or rapid turnover since the divergence of the species. The neighbor-joining tree was generated using the JTT substitution matrix and a gamma rate distribution (see Methods for details).

10.7717/peerj.13954/supp-17 Supplemental Information 17 Comparative plot of within-pool polymorphism on scaf6 Comparative plot of polymorphism on scaf6 within the San Juan River (SJR) and US Environmental Protection Agency (EPA) genomic pools (see Methods for pool descriptions). A narrow peak of within-pool polymorphism (average minor allele frequency in 50-kb windows) is seen in the US Environmental Protection Agency (EPA) population pool within the *vwa3a* gene, whereas no peak is associated with the *ptprua* gene.

## Additional Information and Declarations

### Field Study Permissions

The following information was supplied relating to field study approvals (i.e., approving body and any reference numbers): All activities were performed by state and federal employees authorized to do so by their agencies. Sampling was authorized under a State of Missouri Wildlife Collector’s Permit (#19224) issued to R. Hrabik.

### Data Availability

The following information was supplied regarding data availability: The raw short-read data for this study is available at NCBI: PRJNA806159 and PRJNA806850. The newly assembled mitogenomes for the *P. tenellus* and *P. notatus* samples are available at NCBI: OM718772–OM718774 and OM743017.
