# Supplementary figures and images for: Genome resequencing clarifies phylogeny and reveals patterns of selection in the toxicogenomics model Pimephales promelas
Source: PeerJ. 2022 Aug 25;10:e13954. doi: 10.7717/peerj.13954 (PMC9420404; doi:10.7717/peerj.13954)

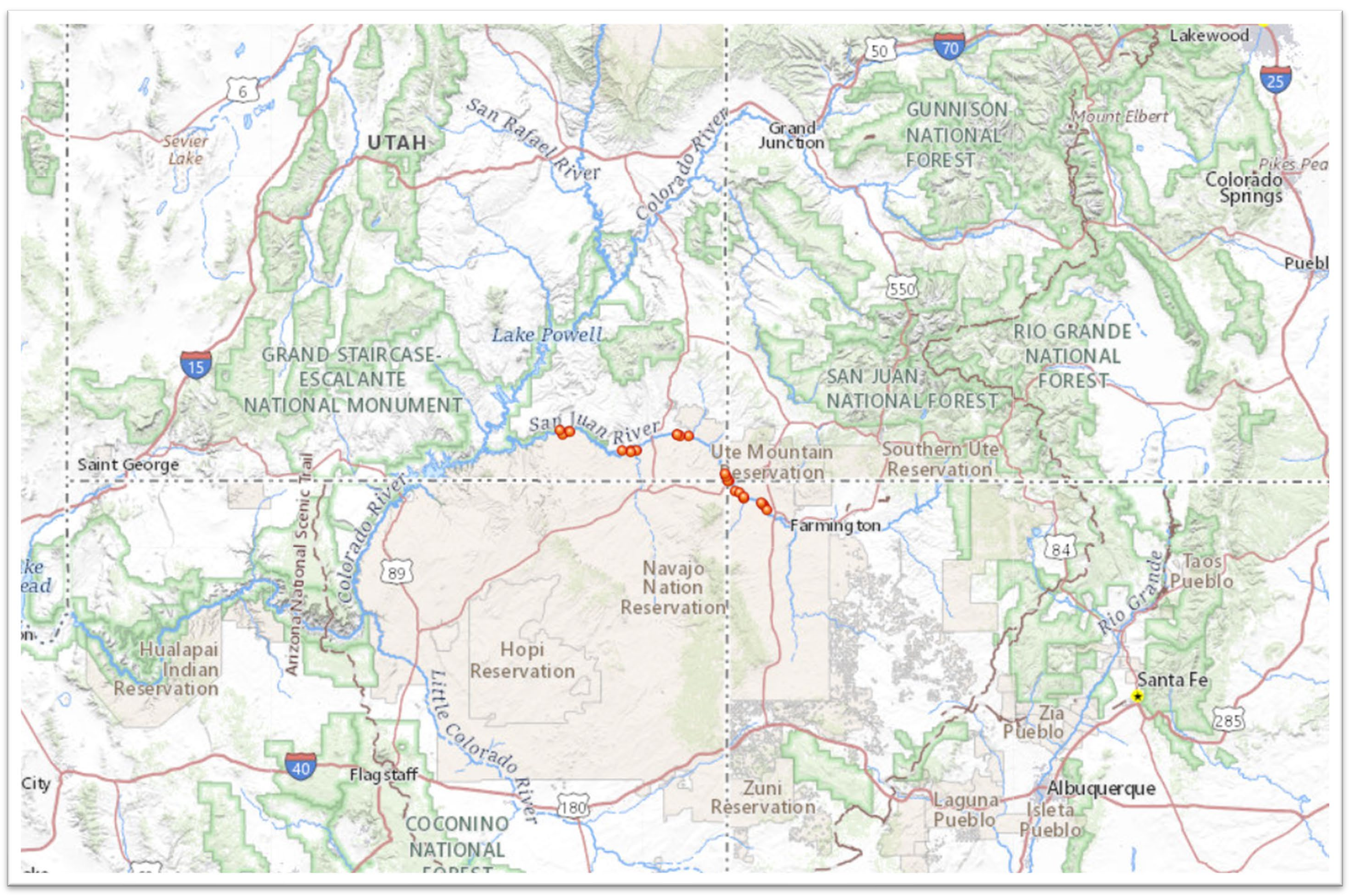

Supplement: Supplemental Information 2 — Map generated with the National Map Viewer tool (https://apps.nationalmap.gov/viewer/) using coordinates provided by the Museum of Southwestern Biology, Albuquerque, New Mexico. [file peerj-10-13954-s002.png]

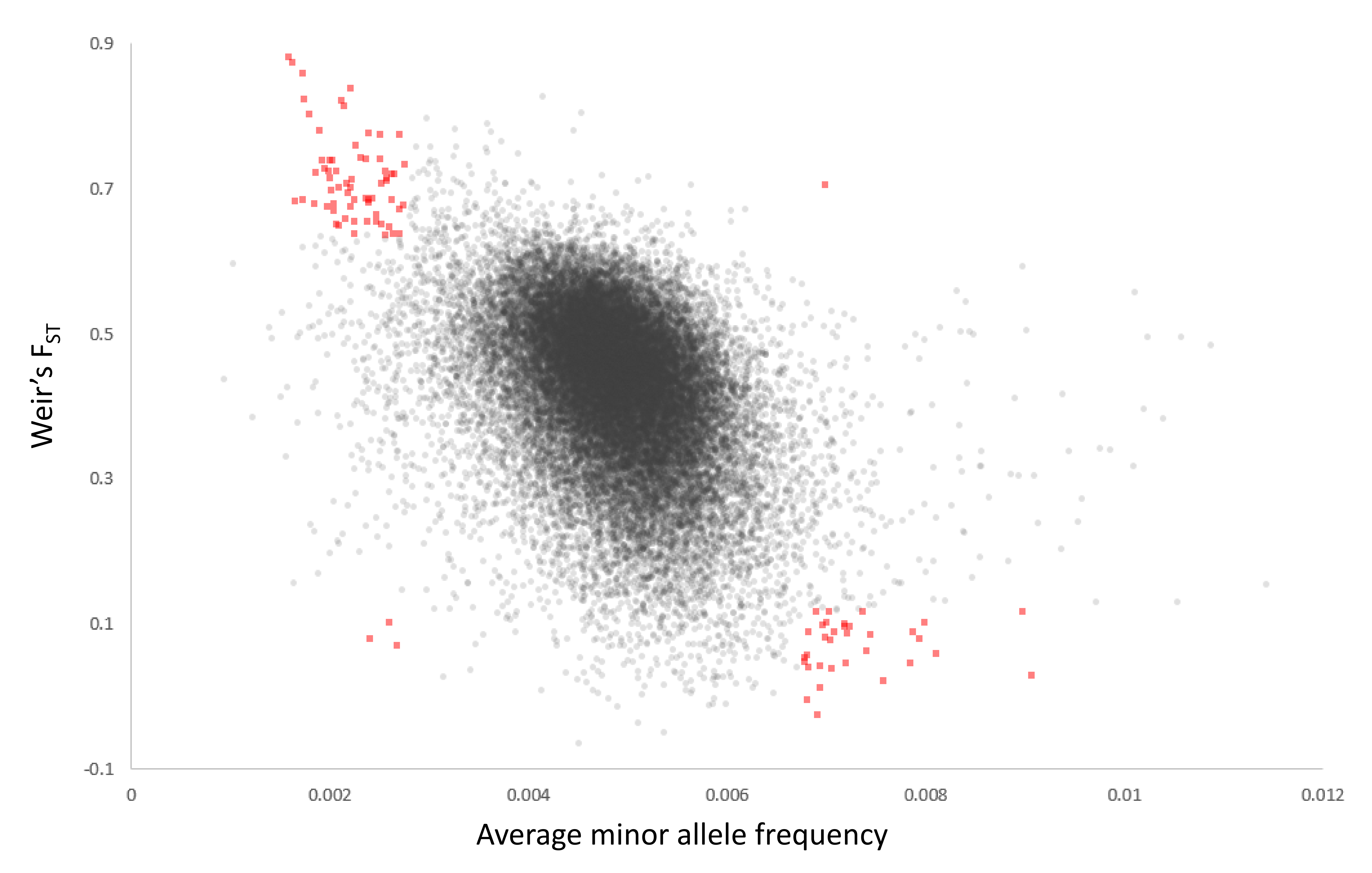

Supplement: Supplemental Information 4 — Windows in the 1% or 99% quantiles of both distributions are marked red. [file peerj-10-13954-s004.png]

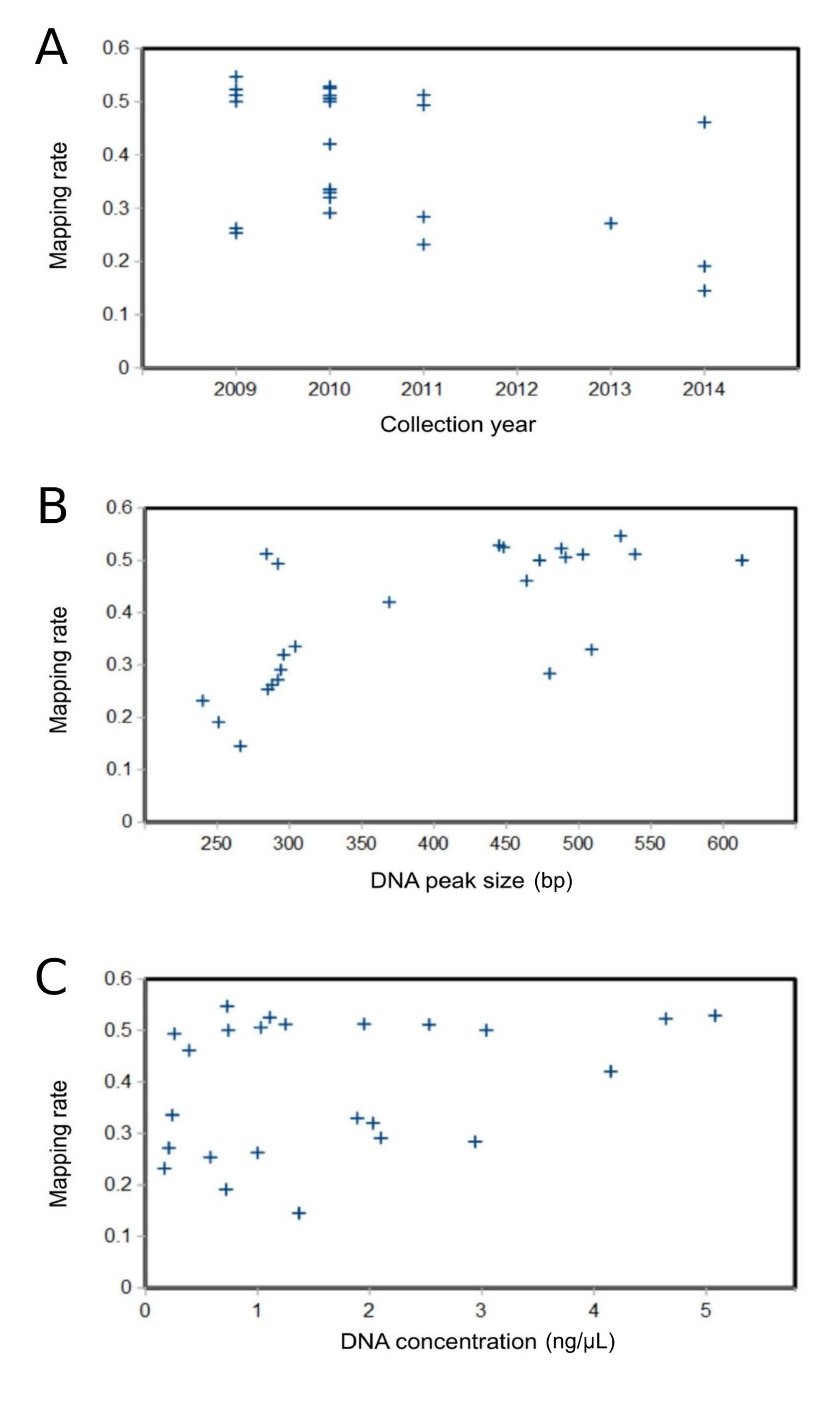

Supplement: Supplemental Information 6 — (A) Year of sample collection. (B) Library peak fragment size. (C) Extracted DNA concentration. [file peerj-10-13954-s006.png]

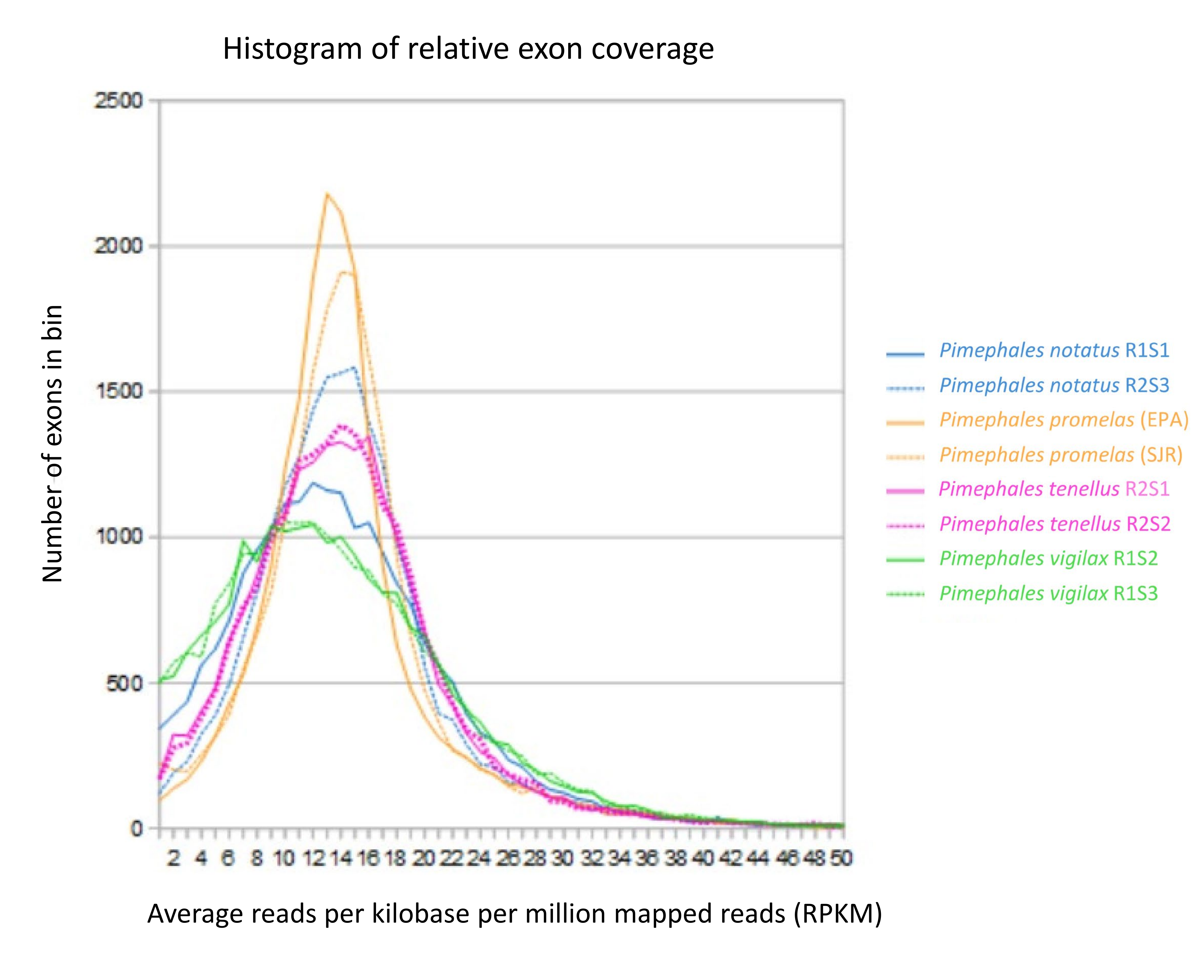

Supplement: Supplemental Information 8 — Horizontal axis represents length-normalized coverage in reads per kilobase per million mapped reads (RPKM). Vertical axis represents the number of exons in each bin of RPKM. [file peerj-10-13954-s008.png]

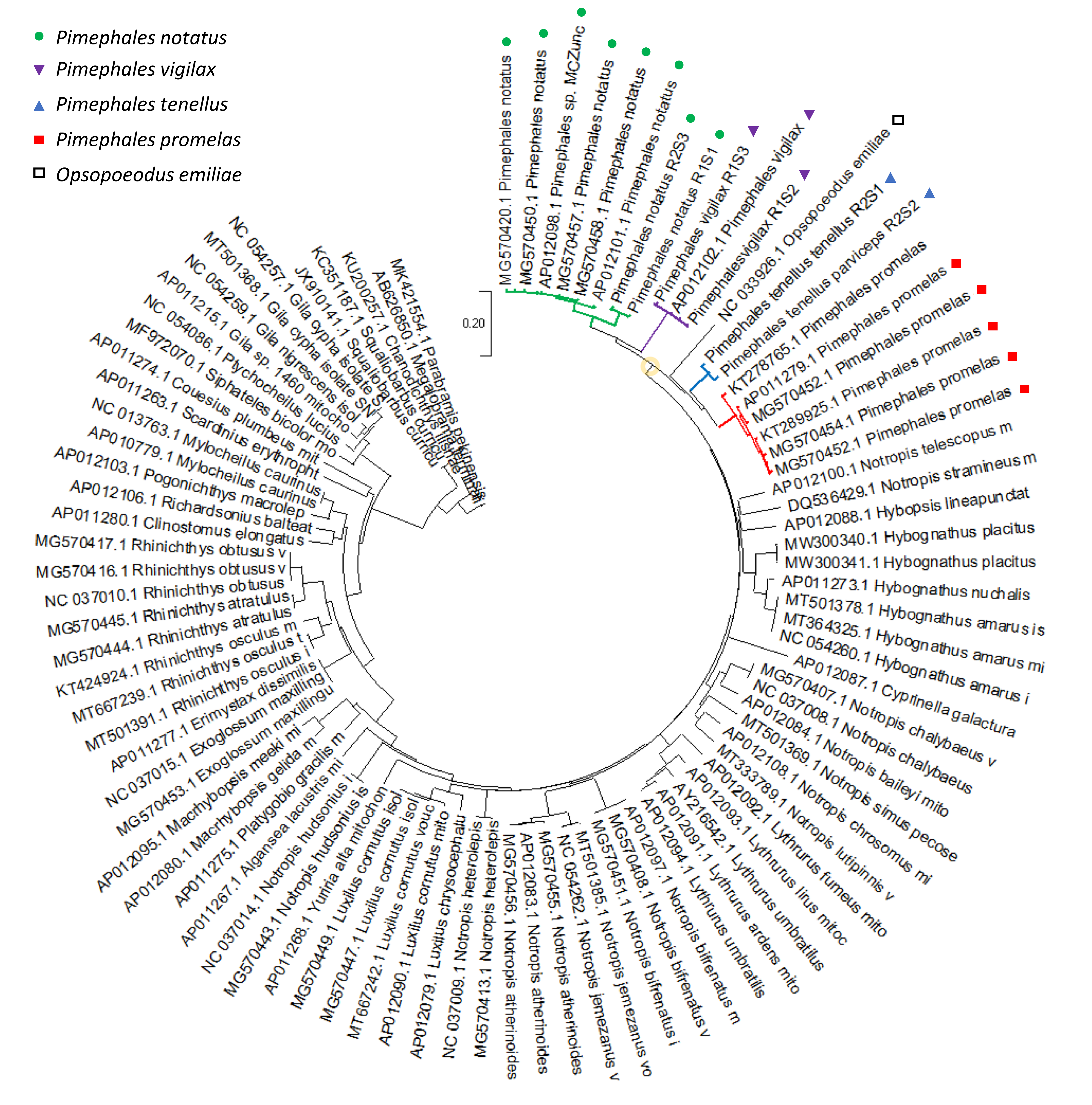

Supplement: Supplemental Information 9 — ¡!–[if !supportAnnotations]–¿¡!–[endif]–¿The basal node of Pimephales plus Opsoepodu s is circled in orange. Branches for each Pimephales species are colored as shown in the legend. ¡!–[if !supportAnnotations]–¿¡!–[endif]–¿ [file peerj-10-13954-s009.png]

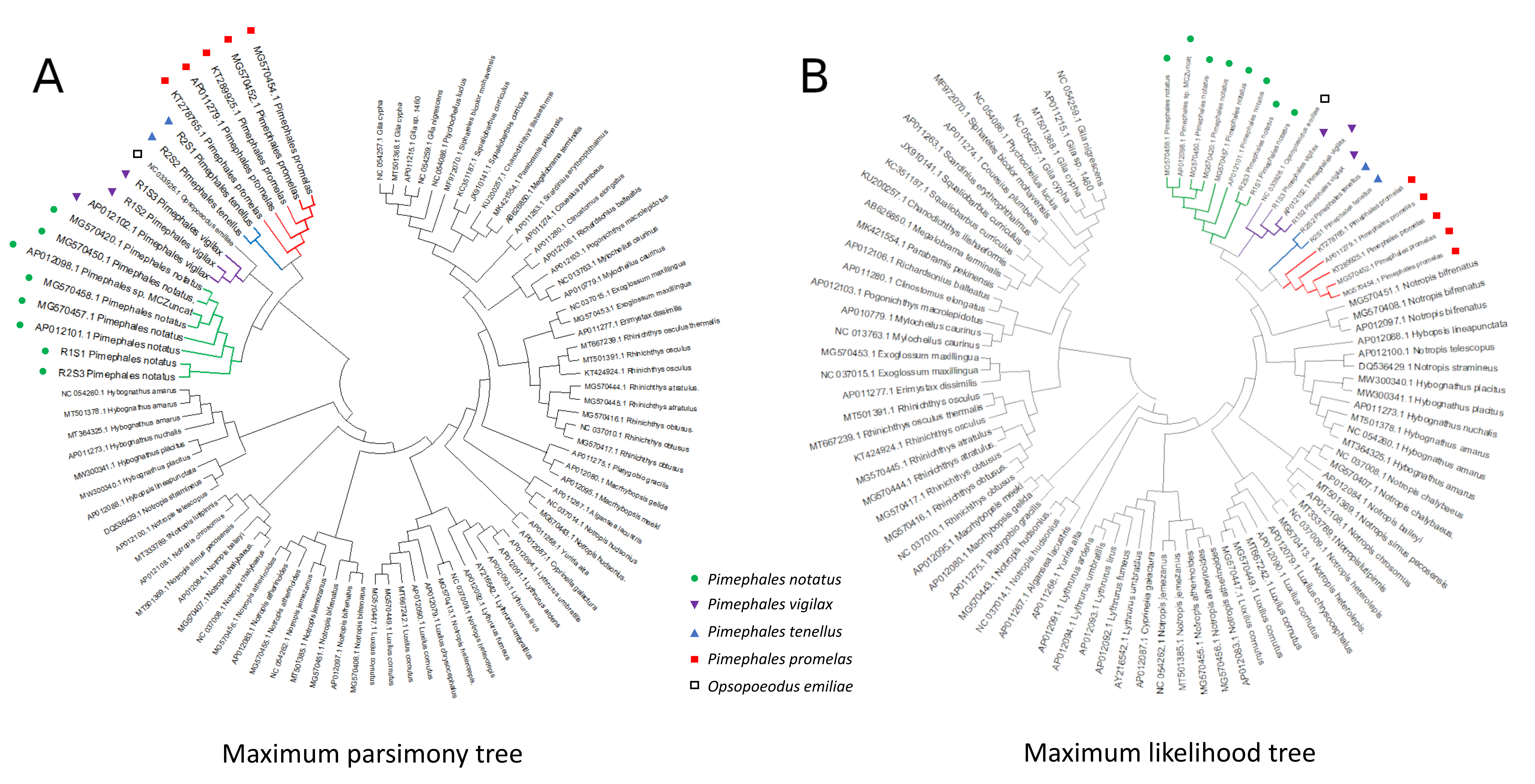

Supplement: Supplemental Information 10 — Phylogenetic topologies recovered for the 92-genome alignment with non-Bayesian methods are identical to the Bayesian tree with respect to the placement of Pimephales and Opsopoeodus. (A) Maximum parsimony phylogeny, and (B) Maximum likelihood phylogeny. See Methods for analysis parameters. [file peerj-10-13954-s010.png]

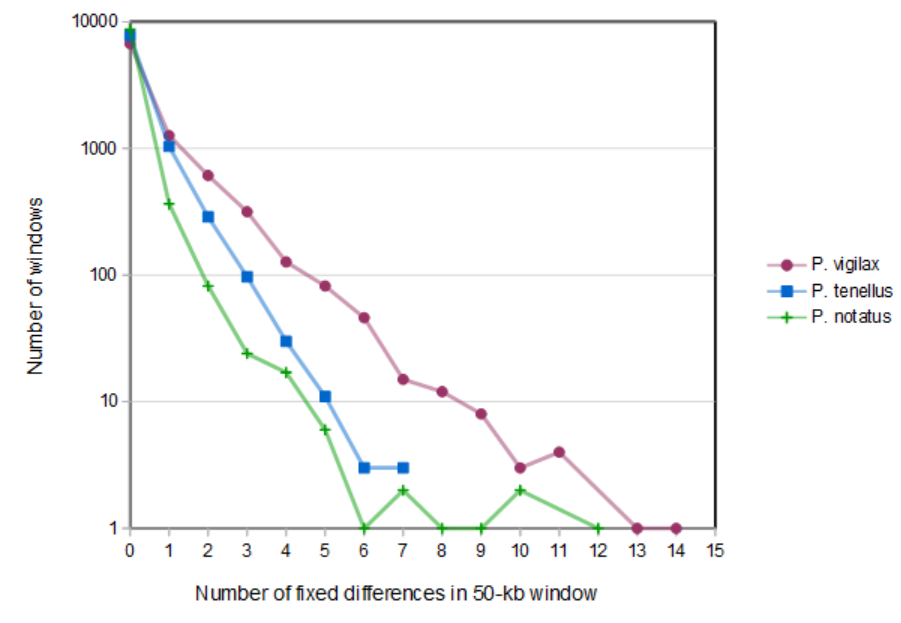

Supplement: Supplemental Information 11 [file peerj-10-13954-s011.png]

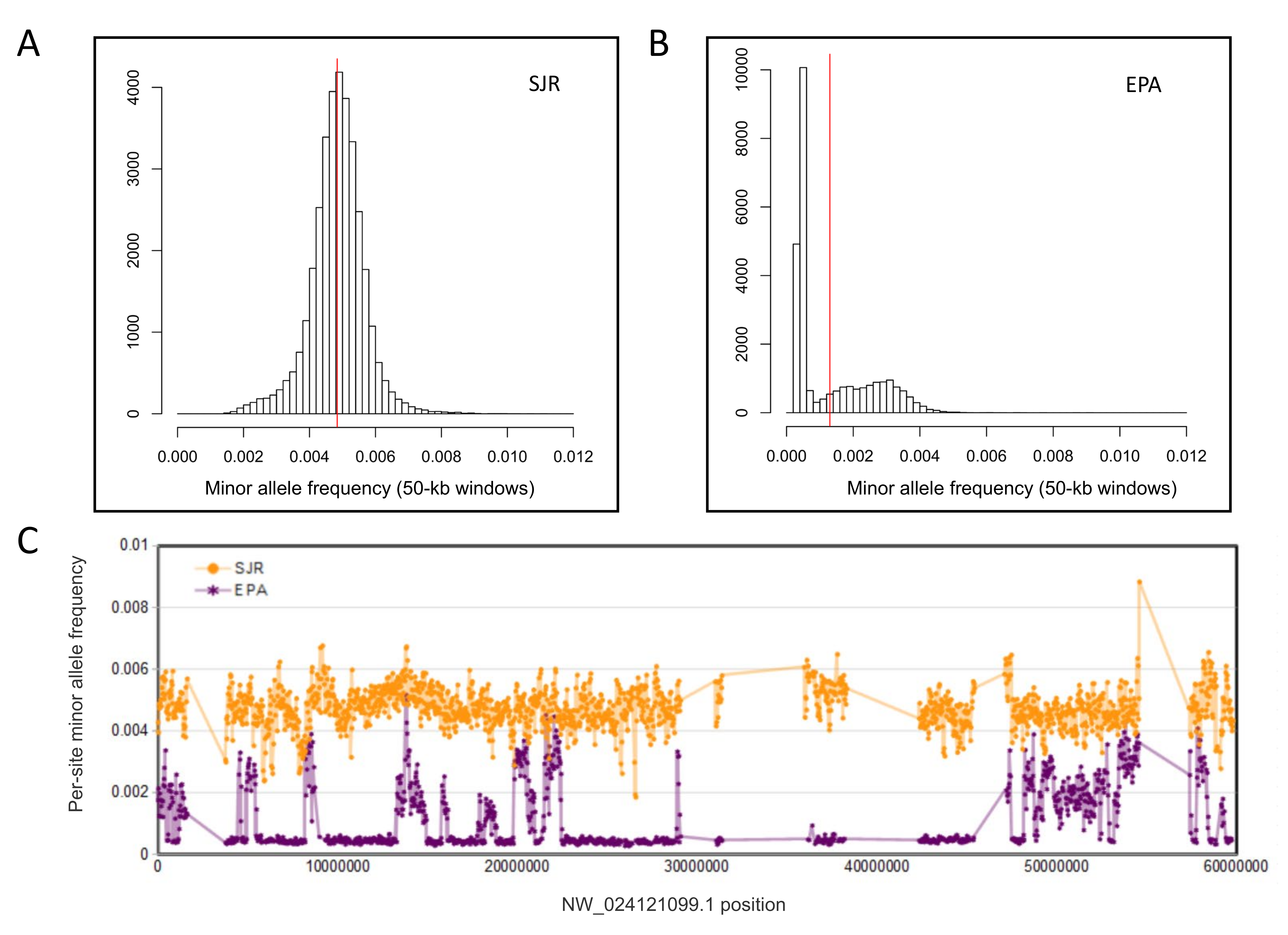

Supplement: Supplemental Information 12 — (A) Histogram of average MAF for SJR genomic windows with the mean value of all windows marked by the red line. (B) Histogram of average MAF for EPA genomic windows with the mean value of all windows marked by the red line. (C) Population MAF in individual genomic windows ordered by position on scaf1, the longest scaffold in the Pimephales promelas assembly, revealing a dichotomous pattern of relatively high or very low MAF in the EPA pool in contiguous genomic regions. [file peerj-10-13954-s012.png]

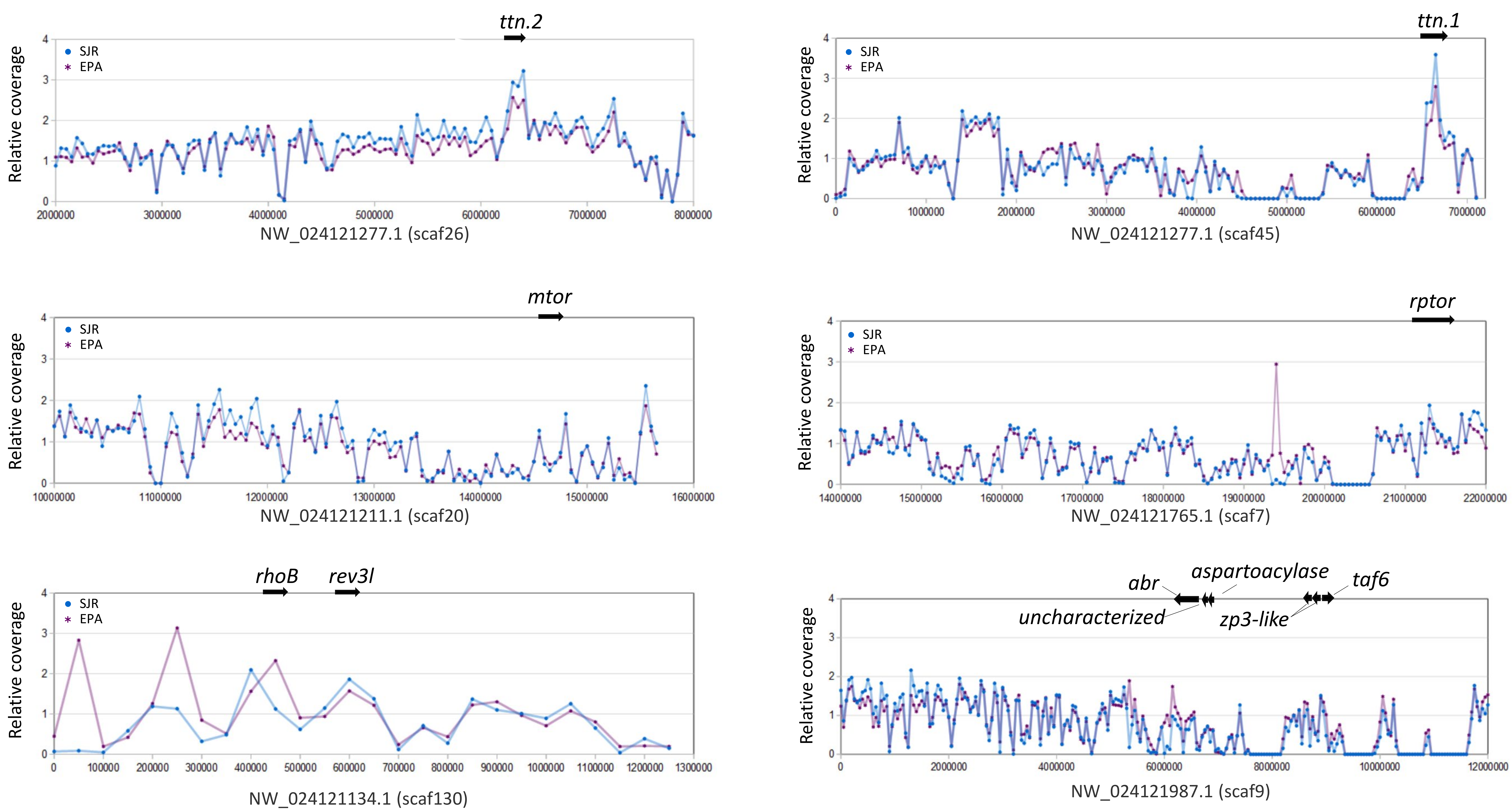

Supplement: Supplemental Information 13 — Depth of mapped reads in non-overlapping 50-kb windows is shown for both Pimephales promelas pools for the regions analyzed in Figs. 2–5. Coverage is scaled relative to the median value of all windows, and is expected to vary due to stochasticity, biased capture of extreme nucleotide compositions, and the proportion of masked or ambiguous sequence in a window (which prevents reads from mapping). Gene-rich regions with few gaps should therefore have somewhat higher than median coverage. Note that dots are positioned according to the first coordinate in each window. [file peerj-10-13954-s013.png]

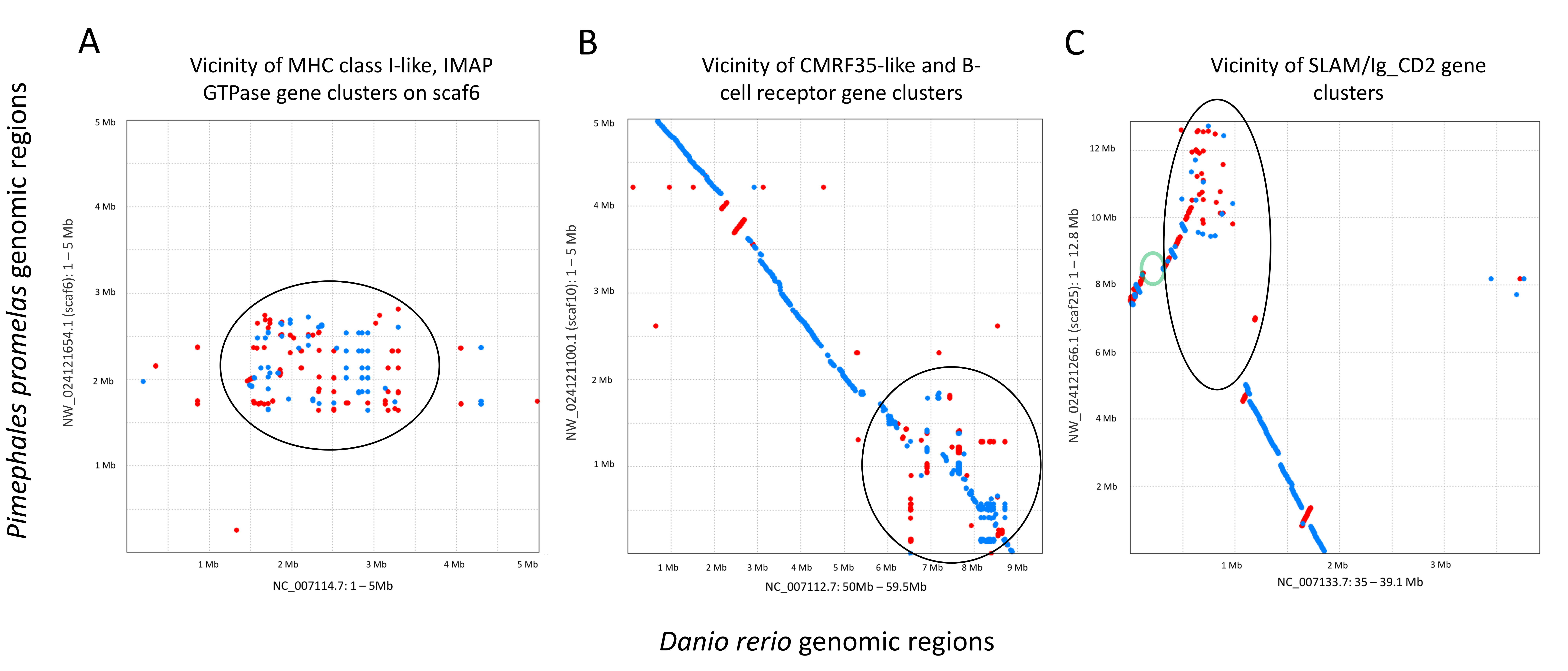

Supplement: Supplemental Information 14 — Each axis represents the genomic sequence of the indicated accession and coordinates. Each dot represents protein homology between the two genome regions that exceeds the scoring threshold. Blue dots indicate homology in the same chromosomal orientation and red dots indicate opposite orientations. Ancestral synteny is indicated by contiguous points forming an approximately diagonal line, inversions as reversals in the orientation and color of the line, and loss of synteny as unordered pattens of pairwise homology or absence of homology. (A–C) show the P. promelas regions containing clusters of immune genes identified in Fig. 6 and their best corresponding match in D. rerio. Equivalent gene clusters in the two species are approximated by black circles. The green circle in (C) highlights a deletion in the P. promelas assembly of a cluster of zinc-finger (ZNF) transcription factors present in D. rerio. [file peerj-10-13954-s014.png]

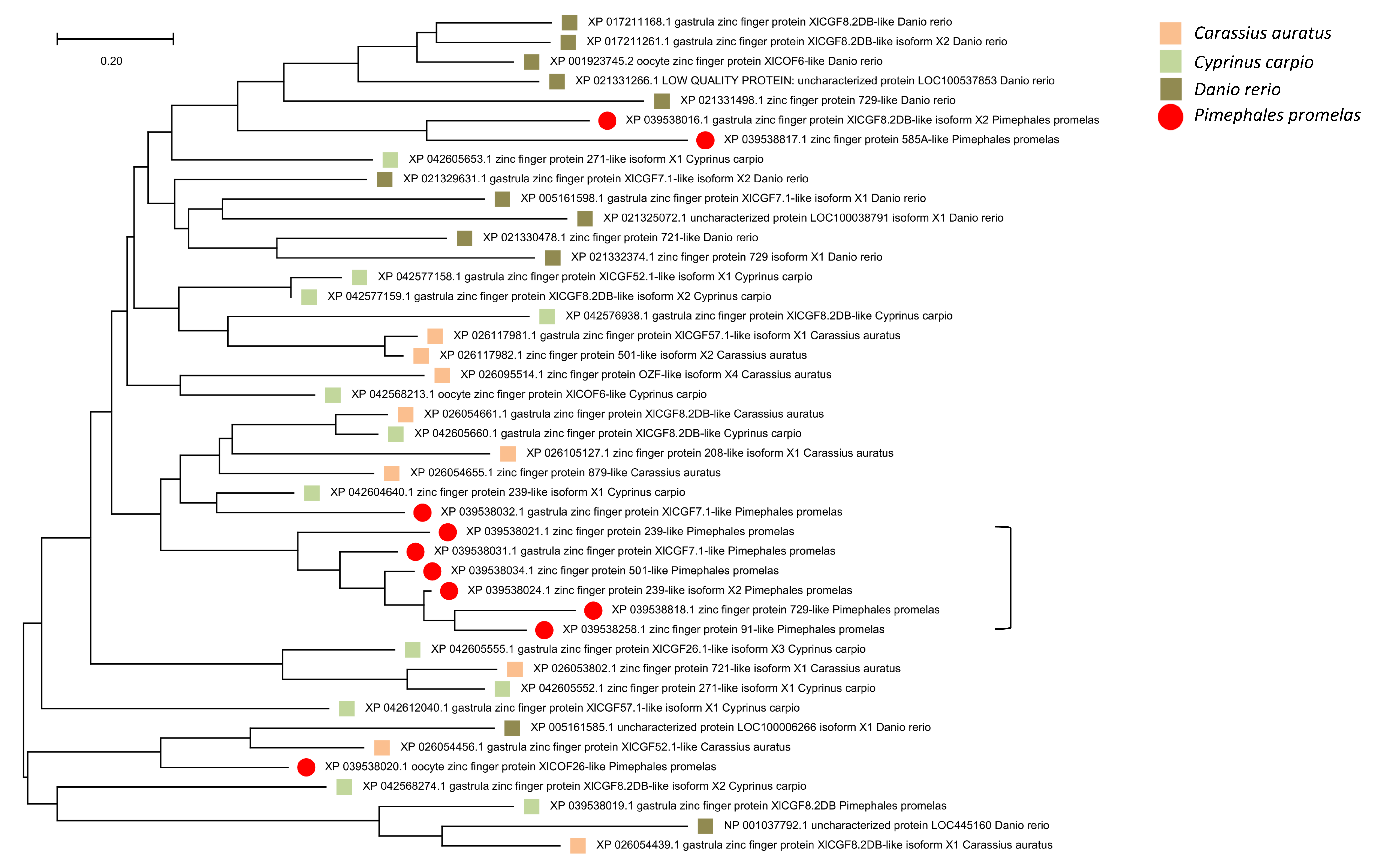

Supplement: Supplemental Information 16 — Gene tree of ten zinc-finger protein (ZNF) transcription factors within the genomic windows marked on scaf25, together with homologs identified in three other high-quality de novo genome assemblies within Cyprinidae: Danio rerio, Carassius auratus, and Cyprinus carpio. (An eleventh Pimephales promelas ZNF gene is adjacent but not within the marked genomic windows). Six of the ten P. promelas genes cluster together with relatively short branches (marked by bracket), indicating gene amplification or rapid turnover since the divergence of the species. The neighbor-joining tree was generated using the JTT substitution matrix and a gamma rate distribution (see Methods for details). [file peerj-10-13954-s016.png]

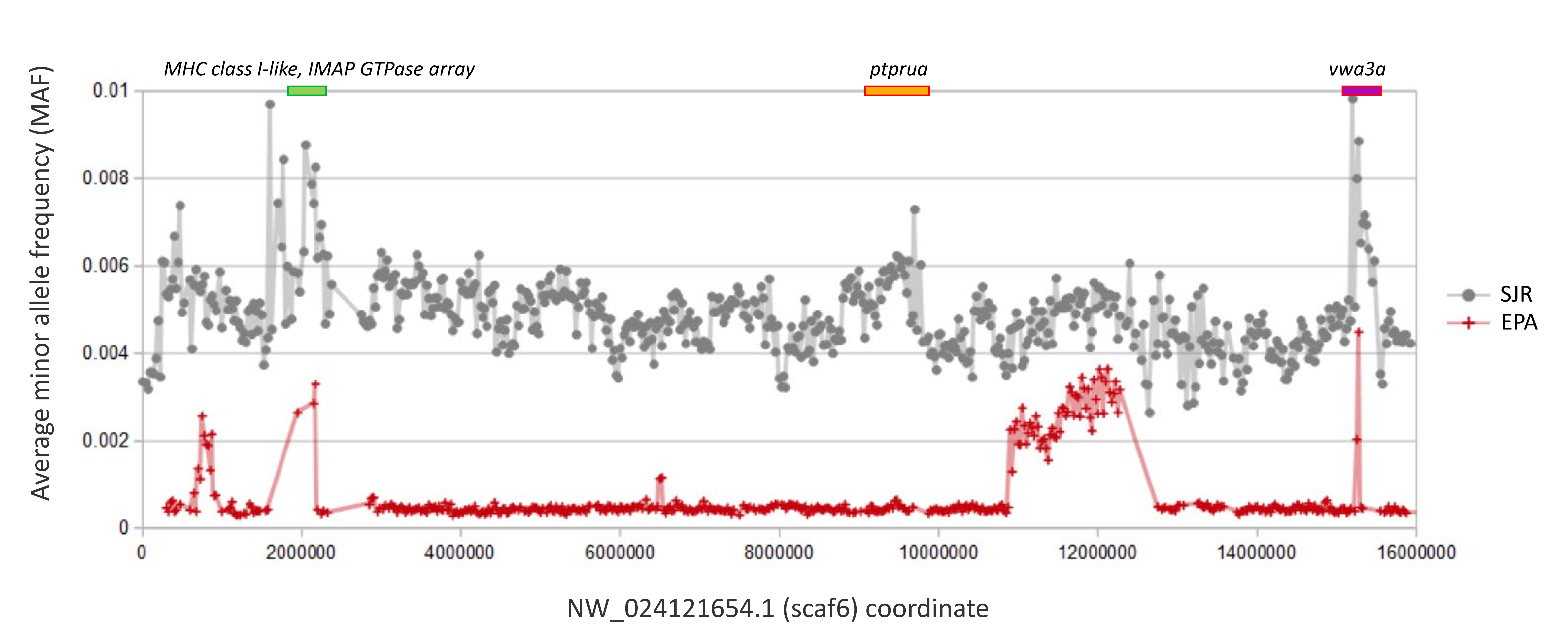

Supplement: Supplemental Information 17 — Comparative plot of polymorphism on scaf6 within the San Juan River (SJR) and US Environmental Protection Agency (EPA) genomic pools (see Methods for pool descriptions). A narrow peak of within-pool polymorphism (average minor allele frequency in 50-kb windows) is seen in the US Environmental Protection Agency (EPA) population pool within the vwa3a gene, whereas no peak is associated with the ptprua gene. [file peerj-10-13954-s017.png]
